# Expression of a Secretable, Cell-Penetrating CDKL5 Protein Enhances the Efficacy of Gene Therapy for CDKL5 Deficiency Disorder

**DOI:** 10.1007/s13311-022-01295-8

**Published:** 2022-09-15

**Authors:** Giorgio Medici, Marianna Tassinari, Giuseppe Galvani, Stefano Bastianini, Laura Gennaccaro, Manuela Loi, Nicola Mottolese, Sara Alvente, Chiara Berteotti, Giulia Sagona, Leonardo Lupori, Giulia Candini, Helen Rappe Baggett, Giovanna Zoccoli, Maurizio Giustetto, Alysson Muotri, Tommaso Pizzorusso, Hiroyuki Nakai, Stefania Trazzi, Elisabetta Ciani

**Affiliations:** 1 Department of Biomedical and Neuromotor Science, University of Bologna, 40126 Bologna, Italy; 2 Department of Developmental Neuroscience, IRCCS Stella Maris Foundation, 56128 Pisa, Italy; 3 Department of Neuroscience, Drug Research and Child Health (NEUROFARBA), University of Florence, 50139 Psychology, Italy; 4 Scuola Normale Superiore, 56126 Pisa, Italy; 5 Departments of Molecular and Medical Genetics and Molecular Immunology and Microbiology Oregon Health & Science University, OR 97239 Portland, USA; 6 Department of Neuroscience “Rita Levi Montalcini”, University of Turin, OR 10126 Turin, Italy; 7 School of Medicine, Department of Pediatrics/Rady Children’s Hospital, University of California San Diego, San Diego, USA; 8 Department of Cellular & Molecular Medicine, Kavli Institute for Brain and Mind, Archealization Center (ArchC), Center for Academic Research and Training in Anthropogeny (CARTA), La Jolla, CA 92037 USA; 9 Institute of Neuroscience, National Research Council, 56126 Pisa, Italy; 10 Division of Neuroscience, Oregon National Primate Research Center, Beaverton, OR 97006 USA

**Keywords:** AAV gene therapy, Cross-correction, CDKL5, Brain disorder, Mouse model

## Abstract

Although delivery of a wild-type copy of the mutated gene to cells represents the most effective approach for a monogenic disease, proof-of-concept studies highlight significant efficacy caveats for treatment of brain disorders. Herein, we develop a cross-correction-based strategy to enhance the efficiency of a gene therapy for CDKL5 deficiency disorder, a severe neurodevelopmental disorder caused by *CDKL5* gene mutations. We created a gene therapy vector that produces an Igk-TATk-CDKL5 fusion protein that can be secreted via constitutive secretory pathways and, due to the cell-penetration property of the TATk peptide, internalized by cells. We found that, although AAVPHP.B_Igk-TATk-CDKL5 and AAVPHP.B_CDKL5 vectors had similar brain infection efficiency, the AAVPHP.B_Igk-TATk-CDKL5 vector led to higher CDKL5 protein replacement due to secretion and penetration of the TATk-CDKL5 protein into the neighboring cells. Importantly, *Cdkl5* KO mice treated with the AAVPHP.B_Igk-TATk-CDKL5 vector showed a behavioral and neuroanatomical improvement in comparison with vehicle or AAVPHP.B_CDKL5 vector-treated *Cdkl5* KO mice. In conclusion, we provide the first evidence that a gene therapy based on a cross-correction approach is more effective at compensating *Cdkl5*-null brain defects than gene therapy based on the expression of the native CDKL5, opening avenues for the development of this innovative approach for other monogenic diseases.

**Supplementary Information:**

The online version contains supplementary material available at 10.1007/s13311-022-01295-8.

## Introduction

CDKL5 (cyclin-dependent kinase-like 5) deficiency disorder (CDD) is a severe X-linked neurodevelopmental disease caused by mutations in the *CDKL5* gene, which lead to a lack of CDKL5 protein expression or function. CDD mainly affects girls and is characterized by early-onset epileptic seizures, hypotonia, intellectual disability, motor and visual impairment, and, in some cases, respiratory dysregulation [1–6]. Although pharmacological treatments are used to control seizures, there is currently no cure or effective treatment to ameliorate cognitive and behavioral symptoms of CDD. The neurological symptoms associated with CDD along with the abundant expression of CDKL5 in the brain [7, 8] suggest that CDKL5 plays a role in brain development and function. Moreover, its localization in mature neurons in both the nucleus and cytoplasm [9–11], implies that CDKL5 plays multiple roles by regulating distinct signaling pathways. Animal models of CDKL5 disorder, *Cdkl5* knockout (KO) mice [12–14], recapitulate different features of CDD, exhibiting severe impairment in learning and memory, visual and respiratory deficits, and motor stereotypies [12, 13, 15–17] and, therefore, they are a good model with which to study the positive effects of therapeutic strategies.

In theory, for a monogenic disease such as CDD, the delivery of a wild-type copy of the mutated gene to cells which lack functional protein represents the most effective approach. Using adeno-associated virus (AAV), advancements in global central nervous system gene delivery have accelerated to the point that treatments for neurodevelopmental disorders, such as lysosomal storage disease, Rett syndrome, CDD, Fragile X, and autism seem within reach [18]. However, gene therapy is not without risks for humans. The major caveat regards the low efficiency of gene delivery to the CNS by viral vectors that requires large vector doses, and consequently, brings the risk of immune reaction, as was the case in human clinical trials for hemophilia B [19, 20]. Moreover, the new gene might be inserted into the DNA in the wrong location, possibly causing harmful mutations to the DNA or even cancer, as shown in rodent studies [21].

We recently developed an enzyme replacement therapy (ERT) based on a recombinant TATk-CDKL5 protein that, when systemically injected, was able to cross the blood–brain barrier and diffuse into brain cells due to the cell-penetration properties of the TATk peptide, retaining its native biological activity [22]. Despite the potential effectiveness of ERT for CDD [22], it requires lifelong treatment with daily administration via an invasive method.

Here, by combining the cell-penetrating property of our recently developed TATk-CDKL5 fusion construct with a secretory Igk-chain leader sequence and the advantages of a gene therapy approach, we have developed a new therapeutic approach that is able to overcome the limitations of both gene therapy and ERT for CDD. The idea behind it is that if the protein produced by the viral vector-infected cells can be secreted and enter into neighboring cells, this will amplify the effect of the gene therapy because, even if the transduced cells are low in number, they will become a “factory” for the production of the therapeutic protein, supplying therapeutic molecules to neighboring cells. In this scenario, the efficiency of gene delivery does not necessarily need to be high. This decreases the risk of insertional mutagenesis and toxic side effects connected with large vector doses while also providing the promise of a life-lasting treatment. We compared the effects of CDKL5 gene therapy with Igk-TATk-CDKL5 gene therapy in a *Cdkl5* KO mouse model to validate whether the Igk-TATk-CDKL5 approach, that enhances the biodistribution of the therapeutic CDKL5 kinase from genetically corrected cells to non-corrected cells via a cross-correction mechanism, significantly enhances therapeutic efficacy.

## Material and Methods

### Cloning of Viral Plasmids and Production of AAV Vectors

To express the Igk-TATk-CDKL5 and CDKL5 proteins by means of AAV vector-mediated gene delivery, the Igk-TATk-hCDKL5_1_ or hCDKL5_1_ gene expression cassettes were subcloned in the backbone of pAAV-CBh-DIO-EGFP (plasmid #87,168, Addgene). The designed viral cassettes between the two AAV2 inverted terminal repeats (ITRs) contain a CBh promoter (0.8 kbp), the Igk-TATk-CDKL5 (3.1 kbp), or CDKL5_1_ (2.9 kbp) open reading frame, followed by a WPRE and an SV40 polyadenylation signal (0.4 kbp). The TATk-CDKL5 and CDKL5 proteins were tagged with a haemagglutinin (HA)-tag. The AAV vector genomes containing the above-described expression cassettes were packaged in the AAVPHP.B capsid in HEK293 cells using an adenovirus-free triple transfection method (for details, see Supplementary material). Igk-TATk sequence: MetETDTLLLWVLLLWVPGSTGDAAQPARRARRTKLAAYARKAARQARA.

### Cell Lines and Primary Cultures

The HEK293T cell line was maintained in Dulbecco modified Eagle medium (DMEM, Gibco) supplemented with 10% heat-inactivated FBS (Gibco), 2 mM of glutamine (Gibco), and antibiotics (penicillin, 100 U/mL; streptomycin, 100 μg/mL; Gibco), in a humidified atmosphere of 5% of CO_2_ at 37 °C. Total cell medium was replaced every 3 days and the cells were sub-cultured once they reached 90% confluence. Cells were transfected with designated plasmid DNA using Metafectene Easy Plus (Biontex). Forty-eight hours after transfection, cells were harvested, washed in PBS, and lysed for total protein extraction. Cell medium was also collected and 200 × concentrated as described previously [22]. Cell extracts and medium were used for Western blot analysis.

Primary hippocampal neuronal cultures were prepared from 1-day-old (P1) wild-type and *Cdkl5* − /Y mice as previously described [23]. Briefly, hippocampi were dissected from mouse brains under a dissection microscope and treated with trypsin (Gibco) for 15 min at 37 °C and DNase I (Sigma-Aldrich) for 2 min at room temperature before being triturated mechanically with a fire-polished glass pipette to obtain a single-cell suspension. Cells were plated on coverslips coated with poly-l-lysine in 6-well plates and cultured in Neurobasal medium (Gibco) supplemented with B27 (Invitrogen) and glutamine (Gibco). Cells were maintained in vitro at 37 °C in a 5% CO_2_-humified incubator.

### In Vitro AAV Transduction

Primary hippocampal neurons were infected with AAVPHP.B_Igk-TATk-CDKL5 and AAVPHP.B_CDKL5 (MOI of 10^6^) at day 2 in vitro (DIV), and fixed at DIV7 with 4% paraformaldehyde + 4% sucrose in 100 mM phosphate buffer pH 7.4. Fixed cells were stained with the primary and secondary antibodies listed in Supplementary Table 1. Nuclei were counterstained with Hoechst-33342 (Sigma-Aldrich) and fluorescent images were acquired using a Nikon Eclipse Te600 microscope equipped with a Nikon Digital Camera DXM1200 ATI system (Nikon Instruments, Inc. Melville, NY, USA).

### Co-culture System

For co-culture experiments, HEK293T cells were plated in a 6-well plate, while primary hippocampal neurons were plated on cover glasses. Twenty-four hours after plating, HEK293T cells were transfected with the AAV vector plasmid containing the Igk-TATk-CDKL5 or CDKL5 cassette. Twenty-four hours after transfection, the co-culture was prepared as follows: HEK293T cells were washed twice with fresh neuronal culture medium. The cover glasses with primary hippocampal neurons (DIV5) were transferred to the HEK293T 6-well plate in an elevated position with respect to the gel supports underneath. After 48 h of co-culturing, the cover glasses with neurons were removed; neurons were washed in PBS, fixed with 4% paraformaldehyde + 4% sucrose in 100 mM phosphate buffer pH 7.4 and processed for immunocytochemistry. The primary and secondary antibodies used are listed in Supplementary Table 1.

### Animal Husbandry

The mice used in this work derive from the *Cdkl5* − /Y strain in the C57BL/6 N background developed in [13] and backcrossed in C57BL/6 J for three generations. Animals were genotyped as previously described [13]. Age-matched wild-type (+ /Y) littermates were used for all experiments. The day of birth was designated as postnatal day (P) zero and animals with 24 h of age were considered as 1-day-old animals (P1). Mice were housed 3–5 per cage on a 12-h light/dark cycle in a temperature- (23 °C) and humidity-controlled environment with standard mouse chow and water ad libitum. The animals’ health and comfort were controlled by the veterinary service. All research and animal care procedures were performed in accordance with the Italian and European Community law for the use of experimental animals and were approved by Bologna University Bioethical Committee. All efforts were made to minimize animal suffering and to keep the number of animals used to a minimum.

### In Vivo AAV Delivery

#### Intraventricular Infusion

Neonatal injections were carried out as previously described [24]. 10^11^ viral vector genomes were injected into 1-day post-gestation (P1) neonatal *Cdkl5* − /Y mice via intracerebroventricular injection targeting the anterior horn of the lateral ventricle. Prior to the procedure, pups were incubated on ice for 1 min and subsequently injected using a 33-gauge needle (Hamilton, Reno, NV, USA). Injected neonates were subsequently returned to the dam. Mice were sacrificed 2 months (P60) post-injection.

#### Intracarotid Infusion

Surgery was performed under general anesthesia (ISOFLO, Esteve Spa, 1.8–2.4% in oxygen, inhalation route) with the mouse’s body temperature maintained at 37 °C by using a heating pad and intra-operative analgesia (10 µL of Norocarp dissolved in 1 mL of saline; 0.2 mL subcutaneously, Pfizer). All procedures were performed in sterile conditions. The tip of the catheter was flushed with sterile heparin. The isolation of the carotid artery was performed as previously described. Once isolated, two silk suture threads (Softsilk 5–0) were proximally and distally placed around the common carotid to create a free blood flow segment of artery in which to insert the catheter. The proximal thread (posterior, closed to the heart) was permanently knotted and tied while the distal thread (near the bifurcation of the common carotid) was pulled by another operator to temporarily create an artery segment with no blood flow. With this approach, it was possible to cut the artery, avoiding any bleeding, and to insert the catheter for the infusions. The catheter was inserted between the suture threads, close to the proximal thread, through a hole made by a 90° bent needle (25 G) (a similar approach was documented in [25]). The distal thread was then released, and the viral solution was infused at 50 µL/min speed by using an infusion pump (Harvard Apparatus, Holliston, MA, USA). Mice were injected with a dose of 10^12^ vg/mouse. When all the amount of the solution (200 µL) was infused, the infusion was stopped, and the catheter was gently pulled out. The threads were both tied to prevent bleeding. Permanent occlusion of one common carotid is a protocol largely used in mice to directly inject drugs into the brain with no long-term consequences for the animals. Indeed, the contralateral carotid and the Willis circle granted a physiological cerebral blood flow as previously documented [26]. Finally, suture stitches and an antiseptic ointment (Betadine 10%, Viatris) were applied to the skin incision. At the end of the surgical procedure, an antibiotic solution (30 μL of Veterinary Rubrocillin, Intervet, Schering-Plow Animal Health, dissolved in 0.8 mL of sterile saline) was administered subcutaneously to prevent infections and to rehydrate the animal.

### Behavioral Testing

Behavioral tests were performed 2 months after the intracarotid infusion. The sequence of the tests was arranged to minimize the possibility of one test influencing the subsequent evaluation of the next test, and mice were allowed to recover for 2 days between different tests. Mice were allowed to habituate to the testing room for at least 1 h before the test, and testing was always performed at the same time of day. Behavioral studies were carried out on the saline-injected *Cdkl5* + /Y and *Cdkl5* − /Y mice groups and AAV-injected *Cdkl5* − /Y mice groups and animals were randomized into each group. Three independent animal cohorts, temporally scaled to allow behavioral tests to be performed in the same succession, were used, for a total of 87 animals. The first test cohort consisted of 34 animals (*Cdkl5* + /Y + vehicle *n* = 10, *Cdkl5* − /Y + vehicle *n* = 6, *Cdkl5* − /Y + AAVPHP.B_CDKL5 *n* = 10, and *Cdkl5* − /Y + AAVPHP.B_Igk-TATk-CDKL5 *n* = 8) that were tested with the following assays: marble burying, nesting test, hindlimb clasping, open field, Barnes maze. The second cohort consisted of 34 animals (*Cdkl5* + /Y + vehicle *n* = 4, *Cdkl5* − /Y + vehicle *n* = 10, *Cdkl5* − /Y + AAVPHP.B_CDKL5 *n* = 10, and *Cdkl5* − /Y + AAVPHP.B_Igk-TATk-CDKL5 *n* = 10) that were tested with the following assays: marble burying, nesting test, hindlimb clasping, open field. The third cohort consisted of 19 animals (*Cdkl5* + /Y + vehicle *n* = 6, *Cdkl5* − /Y + AAVPHP.B_CDKL5 *n* = 6, and *Cdkl5* − /Y + AAVPHP.B_Igk-TATk-CDKL5 *n* = 7) that were tested with the following assays: marble burying, nesting test, hindlimb clasping, open field, Barnes maze. The behavioral test was performed by operators who were blind to genotype and treatment. See Supplementary material for detailed behavioral methods.

The overall behavioral improvement of *Cdkl*5 − /Y mice subjected to gene therapy with AAVPHP.B_Igk-TATk-CDKL5 or AAVPHP.B_CDKL5 vector was evaluated through an average behavioral score for each genotype and treatment in different tests. Mice were assessed on a 1–4 scale for each behavioral test: marble burying, nesting test, hindlimb clasping, open field (velocity and distance moved), and Barnes maze tests. For each test, the gap between the minimum and maximum values was then divided into four ranges. Scores of 1–4 for each quartile were assigned based on performance increase. We included the 5- to 15-min interval for the open field test (velocity and distance moved) in the global score evaluation.

At sacrifice, a total of 41 animals for the subsequent immunohistochemistry analyses and Golgi staining was randomly selected from two of the three independent animal cohorts; first cohort (*Cdkl5* + /Y + vehicle *n* = 3, *Cdkl5* − /Y + vehicle *n* = 3, *Cdkl5* − /Y + AAVPHP.B_CDKL5 *n* = 4, and *Cdkl5* − /Y + AAVPHP.B_Igk-TATk-CDKL5 *n* = 4), second cohort (*Cdkl5* + /Y + vehicle *n* = 4, *Cdkl5* − /Y + vehicle *n* = 7, *Cdkl5* − /Y + AAVPHP.B_CDKL5 *n* = 8, and *Cdkl5* − /Y + AAVPHP.B_Igk-TATk-CDKL5 *n* = 8).

### Non-Invasive Assessment of Sleep and Breathing Pattern

Hypnic and respiratory phenotypes of mice were assessed non-invasively with a validated technique based on whole-body plethysmography (WBP) [27, 28]. Briefly, 2 months after the intracarotid injection, in the lights on condition, each mouse was placed inside a modified 2-chamber WBP (PLY4223, Buxco, Wilmington, NC, USA) flushed with air at 1.5 L/min. The mouse chamber was modified by inserting a solid, machined 10-cm diameter plexiglas block, which reduced the internal volume to 0.97 L. The mean recording length was 6.41 ± 0.04 h (the range was 5.75–6.84 h). The respiratory (WBP chamber pressure) signal was continuously recorded together with chamber humidity and temperature, digitized, and stored at 128 Hz, 4 Hz, and 4 Hz, respectively. The system was calibrated with a 100 μL micro-syringe (Hamilton, Reno, USA) at the end of each recording. The states of wakefulness, non-rapid-eye-movement sleep (NREMS), and rapid-eye-movement sleep (REMS) were scored based on inspection of the raw WBP signal, with the investigators blind to the animal’s genotype. For each mouse, the 15 min at the beginning and 15 min at the end of each recording session were always discarded in order to exclude the periods in which the investigators entered the recording room to connect or disconnect the WBP system. Quantitative analysis of breathing was restricted to stable sleep episodes ≥ 12 s because of the frequent occurrence of movement artefacts during wakefulness. Apneas were automatically detected as breaths with instantaneous total breath duration (TTOT) > 3 times; the average TTOT for each mouse and sleep state, and detection accuracy were checked on raw recordings. The script for the automatic detection of breaths was internally developed by our group using MATLAB software (MathWork, MA, USA); this has already been used for several other publications [27, 28]. For each mouse, apnea occurrence rate was calculated and reported as normalized values, that is, the number of episodes (apneas) divided by the total time spent either in NREMS or REMS (Supplementary Fig. 4b).

### Assessment of Visual Responses

We measured cortical responses to visual stimulation by using intrinsic optical signal (IOS) imaging. The methods employed in [29] were used. Briefly, in anesthetized mice, the scalp was removed, and the skull carefully cleaned with saline. The skin was secured to the skull using cyanoacrylate. Then a thin layer of cyanoacrylate was poured over the exposed skull to attach a custom-made metal ring centered over the binocular visual cortex. The ring was used to head-fix animals to a magnetic holder during imaging sessions, thus removing motion artifacts due to breathing or heartbeat. After surgery, the animals were left to recover for at least 1 week and then injected intracarotidally with a dose of 10^12^ vg/mouse. Non-invasive transcranial IOS recordings were performed 45 days later under isoflurane anesthesia (0.5–1%), supplemented with an intraperitoneal injection of chlorprothixene hydrochloride (1.25 mg/kg), allowing us to use a lower level of isoflurane anesthesia during imaging to maintain a lightly anesthetized state. Images were obtained using an Olympus microscope (BX50WI). Red light illumination was provided by 8 red LEDs (625 nm, Knight Lites KSB1385-1P) attached to the objective (Zeiss Plan-NEOFLUAR 5x, NA: 0.16) using a custom-made metal LED holder. Visual evoked responses were quantitatively measured as reported in [29].

### CDKL5 mRNA and Protein Detection

Mice were perfused with 4% paraformaldehyde in 100 mM phosphate buffer (pH 7.4). Brains were collected and cut along the midline. Hemispheres were submerged in 4% paraformaldehyde in 100 mM phosphate buffer (pH 7.4) for 24 h at 4 °C and then let sink in sucrose 15%, before being frozen at – 80 °C. The hemispheres were then cut with a cryostat into 15-µm-thick sagittal sections which were serially collected on glasses. The in situ hybridization (ISH) for the *CDKL5* RNA was performed with the Base Scope^®^ technology (Biotechne) following the manufacturer’s protocol using a 1ZZ probe designed on the CDKL5 exon 4. For double staining, the ISH was followed by immunohistochemistry for CDKL5 protein detection.

For immunohistochemistry, brain sections were incubated overnight at 4 °C with a primary anti-HA antibody (Supplementary Table 1) and for 2 h with an HRP-conjugated anti-rabbit secondary antibody (Supplementary Table 1). Detection was performed using either the TSA Cyanine 3 Plus or the TSA Plus Fluorescein Evaluation Kits (Perkin Elmer).

### Single-cell Quantification of mRNA Expression

Hoechst and ISH images of the same cell were analyzed using NIS-Elements AR software (Nikon, Japan). An area was traced around each cell comprising the nucleus (identified by Hoechst counterstaining); the intensity of ISH staining, corresponding with the *CDKL5* mRNA signal, was then quantified by determining the sum intensity of all positive (bright) pixels within the area. Approximately 250 cells were analyzed from each slice. Distribution analyses were performed quantifying the percentage of cells expressing *CDKL5* mRNA within different ranges of signal intensity.

### Quantification of CDKL5 mRNA-positive and Cross-corrected Cells

For the quantification of *CDKL5* mRNA and protein-positive cells, two images per section, from the cortex overlying field CA1 and the hindbrain (*n* = 3–4 sections) were acquired using an Eclipse TE 2000-S microscope equipped with a DS-Qi2 digital SLR camera (Nikon Instruments Inc.). All the positive cells present in the image were manually counted using the point tool of the Image Pro Plus software (Media Cybernetics, Silver Spring, MD, USA). The number of *CDKL*5 mRNA + cells was expressed as a percentage of the total number of cells, identified using Hoechst staining. The number of cross-corrected cells was estimated as the difference between the number of CDKL5 protein-positive cells and the number of *CDKL5* mRNA-positive cells, and expressed as a percentage of the total number of infected cells (RNA + cells) or of the total amount of cells.

### Immunohistochemistry Procedures

Mice were sedated with isoflurane (2% pure oxygen) and sacrificed by cervical dislocation. The brains were quickly removed and cut along the midline. One hemisphere was fixed by immersion in a solution of 4% paraformaldehyde in 100 mM phosphate buffer (pH 7.4) for 48 h and subsequently stored in 20% sucrose for another 24 h, before being frozen in dry ice and kept at – 80 °C. The various brain regions of the other hemisphere was dissected, quickly frozen, and used for viral biodistribution analysis. The fixed hemispheres were then cut with a freezing microtome into 30-μm-thick coronal sections which were serially collected in a 96-well plate containing a solution consisting of 30% glycerol, 30% ethylene glycol, and 0.02% sodium azide in 1X PBS. One out of six free-floating sections (*n* = 10–12 sections) from the hippocampal formation was incubated with the primary antibody. The primary and secondary antibodies used are listed in Supplementary Table 1.

Immunofluorescence images, two images per section, were taken with a Nikon Eclipse TE 2000-S inverted microscope, equipped with a Nikon digital camera DS-Qi2 digital SLR camera (Nikon Corp., Kawasaki, Japan). Quantification of Hoechst-positive nuclei and NeuN-positive cells was conducted in the field CA1 of the hippocampus while AIF-1-positive cells were measured and counted in the cortex overlying field CA1.

For morphometric microglial cell analysis, starting from 20 × magnification images of AIF-1-stained slices, AIF-1-positive microglial cell body size was manually drawn using the Image Pro Plus (Media Cybernetics) measurement function and expressed in μm^2^. Approximately 120 microglia cells were analyzed from each sample.

For the density of AIF-1-positive cells, Hoechst-positive nuclei, and NeuN-positive neurons, cells were manually counted using the point tool of the Image Pro Plus software (Media Cybernetics), and expressed as number of cells/mm^3^.

For quantification of PSD-95 immunoreactive puncta, images from the CA1 layer were acquired using a LEICA TCS SL confocal microscope (LEITZ; Leica Microsystems, Wetzlar, Germany; objective 63 × , NA 1.32; zoom factor = 8). All images were acquired with the same gain and exposure time. To ensure that sampling was done at the same level of antibody penetration, all images were captured at a level corresponding to the mid-distance between the upper and lower focus of each section. A frame average process was used to improve image quality: an entire image frame was scanned multiple times and then the data for each point was averaged to reduce noise. Counting was manually carried out using Image Pro Plus software (Media Cybernetics) and fluorescence signals with an area lower than 0.15 µm^2^ were excluded from the evaluation. Three to four sections per animal were analyzed and the number of PSD-95 immunoreactive puncta was expressed per μm^2^.

### Golgi Staining, Neuronal Tracing, and Spine Evaluation

Hemispheres were Golgi-stained using the FD Rapid GolgiStain TM Kit (FD NeuroTechnologies) as previously described [30]. Dendritic trees of Golgi-stained apical dendritic branches of CA1 field neurons were traced using a dedicated software that was custom-designed for dendritic reconstruction (Immagini Computer), interfaced with Image Pro Plus (Media Cybernetics). Dendritic spine density was measured by manually counting the number of dendritic spines. In each mouse, 10–15 dendritic segments (segment length: 10 µm) from each zone were analyzed and spine density was expressed as the total number of spines per 10 µm. Based on their morphology, dendritic spines can be divided into two different categories that reflect their state of maturation: immature spines and mature spines. The number of spines belonging to each class was counted and expressed as a percentage.

### Western Blotting

HEK293T cells transfected with plasmid DNA were lysates in Laemmli buffer supplemented with β-mercaptoethanol, sonicated, and boiled at 95 °C for 10 min. Brains of treated *Cdkl5* − /Y mice were homogenized in ice-cold RIPA buffer supplemented with 1 mM PMSF, and with 1% protease and phosphatase inhibitor cocktail (Sigma-Aldrich). Equivalent amounts of protein were subjected to electrophoresis on a 4–12% Mini-PROTEAN^®^ TGXTM Gel (Bio-Rad) and transferred to a Hybond-ECL nitrocellulose membrane (Amersham—GE Healthcare Life Sciences). The primary and secondary antibodies used are listed in Supplementary Table 1.

### Real-time PCR

Animals were sedated with isoflurane (2% pure oxygen) and sacrificed for cervical dislocation. The brain was quickly removed and the various brain regions were dissected and stored at − 80 °C.

### Viral Biodistribution Analysis

Genomic DNA (gDNA) was extracted from the brain region of interest with the NucleoSpin^®^Tissue Kit (Macherey–Nagel) extraction kit and 100 ng of gDNA was used as a qPCR template. For the quantification of the number of viral copies, a portion of the viral promoter CBh was amplified (Fw 5′-TACTCCCACAGGTGAGCGG-3′, Rev 5′-GGCAGGTGCTCCAGGTAAT-3′). For data normalization, a portion of the mAgouti gene was also amplified (Fw 5′-GGCGTGGTCAGTGGTTGTG-3′, Rev 5′-TTTAGCTTCCACTAGGTTTCCTAGAAA-3′). For data interpolation, the calibration curves for CBh and mAgouti were generated through the quantification of serial dilutions of the AAV vector plasmid and a plasmid containing Agouti fragment, respectively. A master calibration curve for CBh and mAgouti was calculated as the mean of several calibration curves obtained from multiple runs (*n* = 9, for CBh; *n* = 12, for mAgouti) [31]. Ct values were interpolated in the master calibration curve to obtain the number of viral copies for each sample and the mAgouti copy number for internal normalization. Finally, the viral copy number/cell was calculated for each sample considering the murine genomic DNA molecular weight of 3 pg (6 pg per diploid cells).

### Analysis of AAV Vector Genome Transcripts

Total RNA was isolated from the brains of *Cdkl5* − /Y mice treated with vehicle, AAVPHP.B_CDKL5, or AAVPHP.B_Igk-TATk-CDKL5 with the GRS FullSample Purification Kit (GRISP) according to the manufacturer’s instruction. Isolated mRNA was subjected to a DNase I treatment (GRISP), and cDNA synthesis was achieved using iScript™ Advanced cDNA Synthesis Kit (Bio-Rad), according to the manufacturer’s instruction. Reverse transcriptase PCRs were performed using SsoAdvanced Universal SYBR Green Supermix (Bio-Rad) in iQ5 Real-Time PCR Detection System (Bio-Rad). A portion of the m-h*CDKL5* (Fw 5′-CTTAAATGCAGACACAAGGAAACAC-3′, Rev 5′-CGAAGCATTTTAAGCTCTCGT-3′) sequence was amplified for the quantification of AAV vector genome transcripts. A portion of m*GAPDH* (Fw 5′-GAACATCATCCCTGCATCCA-3′, Rev 5′-CCAGTGAGCTTCCCGTTCA-3′) sequence was amplified for data normalization. The differential folds of expression were calculated using the ΔΔCt method. Values were expressed as the fold increase in the CDKL5 expression in the cortex relative to that of the wild types.

### Statistical Analysis

Results are presented as mean ±  standard error of the mean (± SE), and *n* indicates the number of mice. Statistical analysis was performed using GraphPad Prism software (GraphPad Software, Inc., San Diego, CA). All datasets were analyzed using the ROUT method (*Q* = 1%) for the identification of significant outliers and the Shapiro–Wilk test for normality testing. Datasets with normal distribution were analyzed for significance using Student’s *t* test or an ordinary one-way analysis of variance (ordinary one-way ANOVA). Post hoc multiple comparisons were carried out using the Fisher least significant difference (Fisher’s LSD) or a Tukey test. Datasets with non-parametric distribution were analyzed using the Kruskal–Wallis test. Post hoc multiple comparisons were carried out using Dunn’s multiple comparison test. For the open field and the learning phase of the Barnes maze, statistical analysis was performed using a repeated measure two-way analysis of variance (RM two-way ANOVA). A probability level of *P* < 0.05 was considered to be statistically significant. A descriptive statistic of the treatment factor is given in the Supplementary Table 2.

## Results

### Production of an AAVPHP.B_Igk-TATk-CDKL5 Vector for the Expression of a Secretable TATk-CDKL5 Protein

**Fig. 1:**
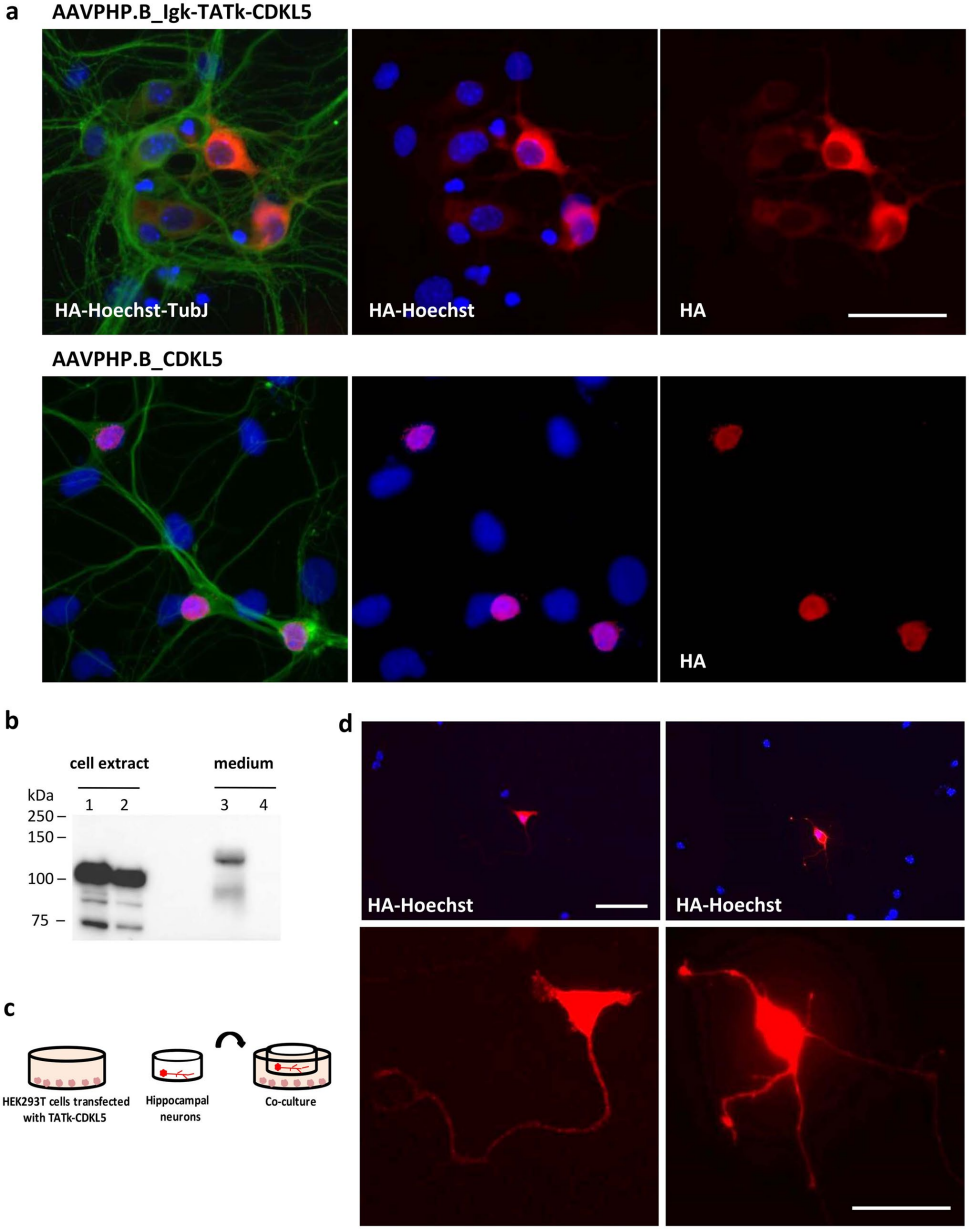
Secretion and transduction efficiency of the TATk-CDKL5 protein. **a** Two-day (DIV2) primary hippocampal neuronal cultures from *Cdkl5* − /Y mice were infected with the AAVPHP.B_Igk-TATk-CDKL5 or the AAVPHP.B_CDKL5 vector (MOI 10^6^) and fixed at DIV 7. CDKL5 and TATk-CDKL5 protein localization was assessed by immunostaining with an anti-HA antibody (red) and an anti-β III tubulin antibody (TubJ, green). Nuclei were counterstained with Hoechst. Scale bar = 30 µm. **b** Western blot analysis using an anti-HA antibody confirmed TATk-CDKL5 and CDKL5 protein expression in AAV vector plasmid DNA-transfected HEK293T cells (cell extract, lanes 1 and 2), and TATk-CDKL5 protein accumulation in the concentrated culture medium (lane 3), indicating that the TATk-CDKL5 protein was secreted from cells. No CDKL5 expression was detected in the medium of HEK293T cells transfected with the AAVPHP.B_CDKL5 vector (lane 4). **c** Co-culture experimental design: HEK293T cells were transfected with the AAV vector plasmid containing the Igk-TATk-CDKL5 cassette. Twenty-four hours after transfection, cover glasses with 5-day (DIV5) differentiated primary hippocampal neurons were transferred to the HEK293T 6-well plate in an elevated position. **d** Fluorescence microscopy images showing the presence of TATk-CDKL5 protein in differentiated primary hippocampal neurons from *Cdkl5* − /Y mice co-cultured for 48 h (DIV5-DIV7) with HEK293T cells transfected with AAVPHP.B_Igk-TATk-CDKL5 plasmid. Neurons were immunostained with an anti-HA antibody (red) and nuclei were counterstained with Hoechst. Lower panels, high magnification images of the stained neurons. Scale bars = 70 µm (low magnification), 15 µm (high magnification)

To express the Igk-TATk-CDKL5 and CDKL5 proteins by means of AAV vector-mediated gene delivery, an Igk-TATk-CDKL5 or a CDKL5 gene expression cassette [22] was inserted into the AAV vector plasmid under the control of a strong, long-term, and ubiquitous expression promoter [32]. The ability of the newly generated AAV vectors to express CDKL5 and Igk-TATk-CDKL5 proteins in neurons was confirmed after primary hippocampal neuron infection (Fig. 1a). Igk-TATk-CDKL5 showed a different expression pattern compared to CDKL5, with a more cytoplasmic distribution (Fig. 1a) that suggests the presence of protein secretion via the constitutive secretory pathways. We confirmed efficient TATk-CDKL5 protein secretion using Western blot analysis (Fig. 1b). TATk-CDKL5, but not CDKL5 protein, was detected in the culture medium of HEK293T cells transfected with the AAVPHP.B_Igk-TATk-CDKL5 or the AAVPHP.B_CDKL5 vector plasmid DNA (Fig. 1b). To investigate whether neurons are penetrated by secreted TATk-CDKL5 protein, hippocampal neurons were co-cultured, using transwells (Fig. 1c), with HEK293T cells transfected with the AAV-Igk-TATk-CDKL5 or the AAV-CDKL5 plasmid. After 48 h of co-culture, TATk-CDKL5 protein was efficiently internalized by hippocampal neurons (Fig. 1d), while, as expected, no CDKL5-positive neurons were present (data not shown).

**Fig. 2:**
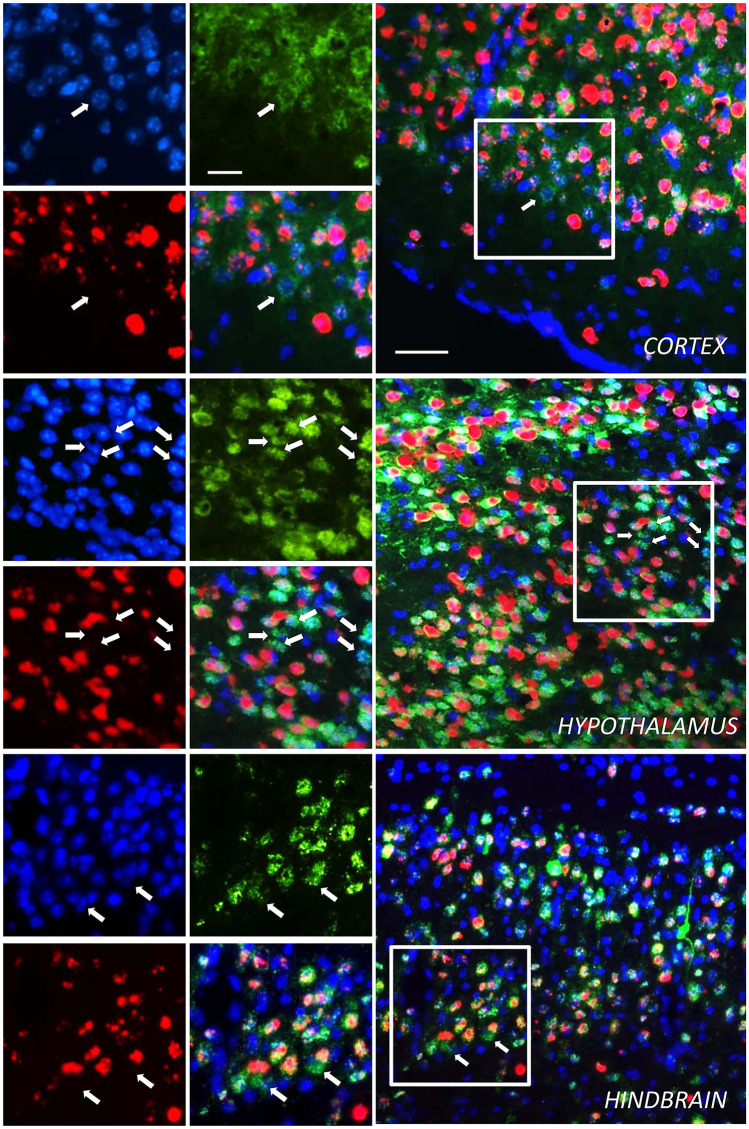
TATk-CDKL5 mRNA and protein distribution in mouse brain sections. Fluorescence in situ hybridization (ISH) for *CDKL5* mRNA combined with fluorescence immunolabeling for TATk-CDKL5 protein in mouse brain sections of 2-month-old *Cdkl5* − /Y mice intraventricularly injected at the neonatal stage with AAVPHP.B_Igk-TATk-CDKL5 vector. Images show *TATk-CDKL5* mRNA (red) and protein (green) localization in the cortex, hypothalamus, and hindbrain of a treated mouse 90 days post-injection. Localization of *TATk-CDKL5* mRNA was evaluated through ISH with a CDKL5 probe, while TATk-CDKL5 protein was evaluated through immunohistochemistry using an anti-HA antibody; nuclei were counterstained with DAPI. The white boxes indicate the regions shown in the high magnification panels. The white arrows indicate cross-corrected cells (HA-immunopositive cells with no ISH staining). Scale bar = 50 µm (low magnification); 25 µm (high magnification)

The efficiency of TATk-CDKL5 protein penetration in vivo was analyzed in *Cdkl5* KO (− /Y) mice that had undergone intraventricular injection with AAVPHP.B_Igk-TATk-CDKL5 vector at the neonatal stage and that were sacrificed 2 months after the injection. By combining fluorescent in situ hybridization (ISH) and immunohistochemical staining to simultaneously visualize *CDKL5* mRNA and protein, respectively, we found CDKL5 protein replacement in brain cells that did not show ISH staining (Fig. 2, Supplementary Fig. 1), indicating a cross-correction mechanism mediated by the secretable, cell-penetrating TATk-CDKL5 protein. By contrast, we did not find brain cells that were positive for CDKL5 protein and that did not show ISH staining in brain slices of AAVPHP.B_CDKL5-treated *Cdkl5* − /Y mice (Supplementary Fig. 2a). Quantification of cross-corrected cells revealed that CDKL5-positive cells that did not show ISH staining made up around 6% of the total infected cells (RNA + cells) in the brain of AAVPHP.B_Igk-TATk-CDKL5-treated *Cdkl5* − /Y mice (Supplementary Fig. 2b), and around 2% of the total cells (data not shown).

### Effect of Gene Therapy on Behavior in Cdkl5 − /Y Mice

To evaluate the effectiveness of a cross-correction mechanism compared to a classic gene therapy approach, adult (3–4 months old) *Cdkl5* − /Y mice were administered with AAVPHP.B_CDKL5 or AAVPHP.B_Igk-TATk-CDKL5 vector at a dose of 10^12^ vg/mouse via intracarotid injection, and the effects of treatment were evaluated 60 days post-injection (Supplementary Fig. 3a). A group of vehicle-treated *Cdkl5* − /Y and wild-type (+ /Y) mice were used as controls for behavioral tests. Importantly, no changes in terms of body weight, sleep pattern, microglial cell number, or cell survival were observed in vector-treated *Cdkl5* − /Y mice compared to age-matched vehicle-treated mice (Supplementary Fig. 3b–d), indicating that viral infection and secreted CDKL5 protein did not affect animal well-being and/or cause an inflammatory response.

**Fig. 3:**
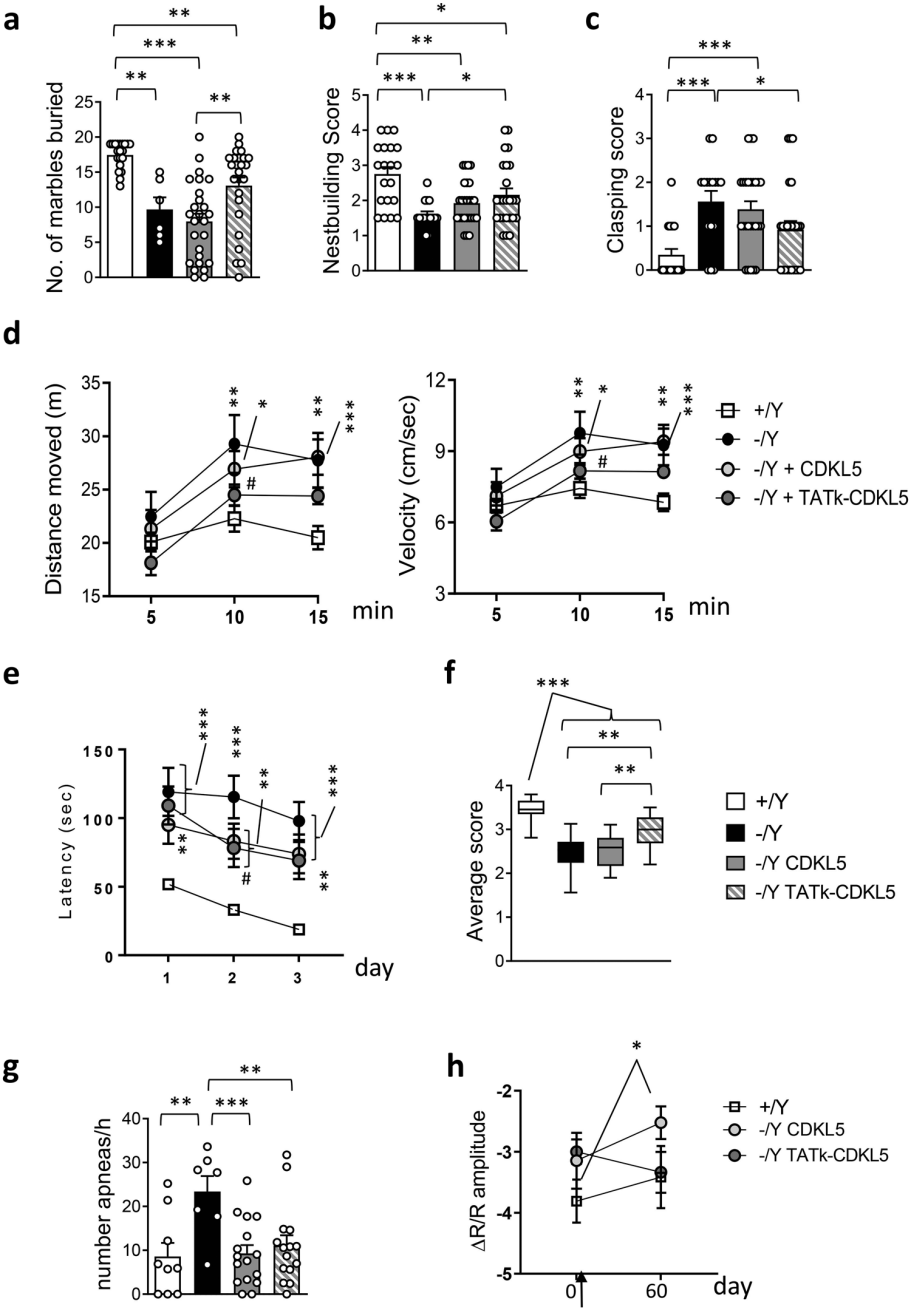
Effect of CDKL5 and TATk-CDKL5 proteins on behavior in *Cdkl5* − /Y mice. **a**, **b** Autistic-like features in treated *Cdkl5* − /Y mice. Number of marbles buried (**a**) and nest quality (**b**) of wild-type mice (+ /Y, *n* = 20) and *Cdkl5* − /Y mice (*n* = 6 (a); *n* = 16 (b)) and of *Cdkl5* − /Y mice 60 days from treatment with AAVPHP.B_CDKL5 (*n* = 26) or AAVPHP.B_Igk-TATk-CDKL5 (*n* = 25). **c** Hind-limb clasping score during a 10-s interval in wild-type mice (+ /Y, *n* = 20) and *Cdkl5* − /Y mice (*n* = 16), and in *Cdkl5* − /Y mice 60 days after treatment with AAVPHP.B_CDKL5 (*n* = 26) or AAVPHP.B_Igk-TATk-CDKL5 (*n* = 25). **d** Total distance traveled (left graph) and average locomotion velocity (right graph) of wild-type mice (+ /Y, *n* = 20) and *Cdkl5* − /Y mice (*n* = 16), and of *Cdkl5* − /Y mice treated with AAVPHP.B_CDKL5 (*n* = 26) or AAVPHP.B_Igk-TATk-CDKL5 (*n* = 25), during a 15-min open field test. **e** Spatial learning was assessed using the Barnes Maze in wild-type mice (+ /Y, *n* = 16) and *Cdkl5* − /Y mice (*n* = 10), and in *Cdkl5* − /Y mice treated with AAVPHP.B_CDKL5 (*n* = 15) or AAVPHP.B_Igk-TATk-CDKL5 (*n* = 13). Graphs show the mean latency to find the target hole during the 3-day learning period. **f** Behavioral score was assessed on a 1–4 scale for marble burying, nesting test, hindlimb clasping, open field (velocity and distance moved), and Barnes maze tests. The average score for each genotype and treatment was calculated. Whiskers show minimum and maximum score values among the same group of animals. **g** Sleep apnea occurrence rate in treated *Cdkl5* − /Y mice was assessed using whole-body plethysmography. Sleep apnea occurrence in vehicle-treated wild-type (+ /Y, *n* = 9) and *Cdkl5* − /Y (*n* = 7) mice, and in *Cdkl5* − /Y mice treated with AAVPHP.B_CDKL5 (*n* = 16) or AAVPHP.B_Igk-TATk-CDKL5 (*n* = 15), during rapid eye movement sleep (REMS). **h** Mean amplitude of visually evoked IOS responses measured before and 60 days after treatment in vehicle-treated (*n* = 7) *Cdkl5* + /Y mice and AAVPHP.B_CDKL5 (*n* = 8) or AAVPHP.B_Igk-TATk-CDKL5 (*n* = 6) treated *Cdkl5* − /Y mice. The black arrow indicates the treatment time, one week after the first visually evoked IOS response measurement. Values (**a**, **b**, **c**, **f**, **g**) are presented as means ± SE. *P < 0.05; **P < 0.01; ***P < 0.001 (datasets in **a**, **b**, **c,** and **f**, Dunn’s test after Kruskal–Wallis; datasets in **g**, Fisher’s LSD test after one-way ANOVA). Values in (**d**, **e**, **h**) are presented as means ± SE. **P < 0.01; ***P < 0.001 compared to the vehicle-treated wild-type condition; #*P* < 0.05 as compared to the vehicle-treated *Cdkl5* − /Y samples (datasets in **d** and **e**, Fisher’s LSD test after RM two-way ANOVA; dataset in **h**, Fisher’s LSD test after two-way ANOVA)

Loss of Cdkl5 function in *Cdkl5* − /Y mice is associated with autistic-like (ASD-like) phenotypes, analyzed through home-cage social behaviors (marble burying and nest building ability) [33]. *Cdkl5* − /Y mice buried a significantly lower number of marbles and showed a reduced nest building ability compared to wild-type (+ /Y) mice (Fig. 3a, b). Sixty days after treatment with AAVPHP.B_Igk-TATk-CDKL5 vector, *Cdkl5* − /Y mice buried a higher number of marbles compared to vehicle-treated and AAVPHP.B_CDKL5-treated *Cdkl5* − /Y mice (Fig. 3a). Similarly, nest building ability was improved only in *Cdkl5* − /Y mice treated with AAVPHP.B_Igk-TATk-CDKL5 vector (Fig. 3b).

Stereotypic movements characterize *Cdkl5* − /Y mice [13, 33] and CDD patients [34]. In order to examine the effect of gene therapy on motor stereotypies, mice were tested for hindlimb clasping (Fig. 3c). Unlike wild-type (+ /Y) mice, vehicle-treated and AAVPHP.B_CDKL5-treated *Cdkl5* − /Y mice showed a higher clasping score (Fig. 3c). On the contrary, *Cdkl5* − /Y mice treated with AAVPHP.B_Igk-TATk-CDKL5 vector showed a decrease in clasping (Fig. 3c), indicating that gene therapy with Igk-TATk-CDKL5 has a greater positive impact on the stereotypic behavior that is due to loss of Cdkl5 expression.

We assessed motor function of treated *Cdkl5* − /Y mice in the open-field test. The elevated locomotor activity (longer distance traveled with a higher average speed; Fig. 3d) that characterizes *Cdkl5* − /Y mice was improved by treatment with AAVPHP.B_Igk-TATk-CDKL5 vector (Fig. 3d), while no improvement was observed in mice treated with AAVPHP.B_CDKL5 vector.

Learning and memory were evaluated using the Barnes maze test, a cognitive paradigm in which *Cdkl5* − /Y mice are documented to be impaired [35]. A significative improvement in learning was observed only in *Cdkl5* − /Y mice treated with AAVPHP.B_Igk-TATk-CDKL5 vector (Fig. 3e). However, no improvement in memory function was observed in *Cdkl5* − /Y mice treated with AAVPHP.B_Igk-TATk-CDKL5 or AAVPHP.B_CDKL5 vector during the probe trial on the 4th day (Supplementary Fig. 4a).

An overall mean phenotype score analysis including ASD-like phenotypes (Fig. 3a, b), stereotypic movements (Fig. 3c), motor function (Fig. 3d), and learning (Fig. 3e) showed a significative improvement only in *Cdkl5* − /Y mice treated with AAVPHP.B_Igk-TATk-CDKL5 vector (Fig. 3f), indicating that gene therapy with Igk-TATk-CDKL5 has a greater positive impact on behavior.

### Effect of Gene Therapy on Breathing Pattern and Cortical Visual Responses in Cdkl5 − /Y Mice

Since impaired breathing pattern, particularly during sleep, and visual responses represent promising biomarkers for preclinical and clinical studies on CDD [22, 28, 29, 33], we evaluated the effect of the treatments with AAVPHP.B_Igk-TATk-CDKL5 and AAVPHP.B_CDKL5 vector on these two patterns in *Cdkl5* − /Y mice. Using whole-body plethysmography, we found that treatment with both AAVPHP.B_CDKL5 and AAVPHP.B_Igk-TATk-CDKL5 vectors led to a drastic reduction in the number of apneas during REM sleep that became similar to that of wild-type mice (Fig. 3g). In contrast, an improvement was not achieved during NREM sleep (Supplementary Fig. 4b).

Cortical visual responses were assessed using non-invasive transcranial intrinsic optical signal (IOS) imaging, a method that allows us to monitor the visually evoked responses in the same animal at different time points [29]. We found that, while AAVPHP.B_CDKL5-treated *Cdkl5* − /Y mice showed a significantly reduced response with respect to the wild-type baseline condition (Fig. 3h), the visual responses of *Cdkl5* − /Y mice treated with the AAVPHP.B_Igk-TATk-CDKL5 vector underwent an improvement, becoming similar to those found in wild-type mice (Fig. 3h).

### Effect of Gene Therapy on Dendritic Hypotrophy and Connectivity in the Hippocampus of Cdkl5 − /Y Mice

**Fig. 4:**
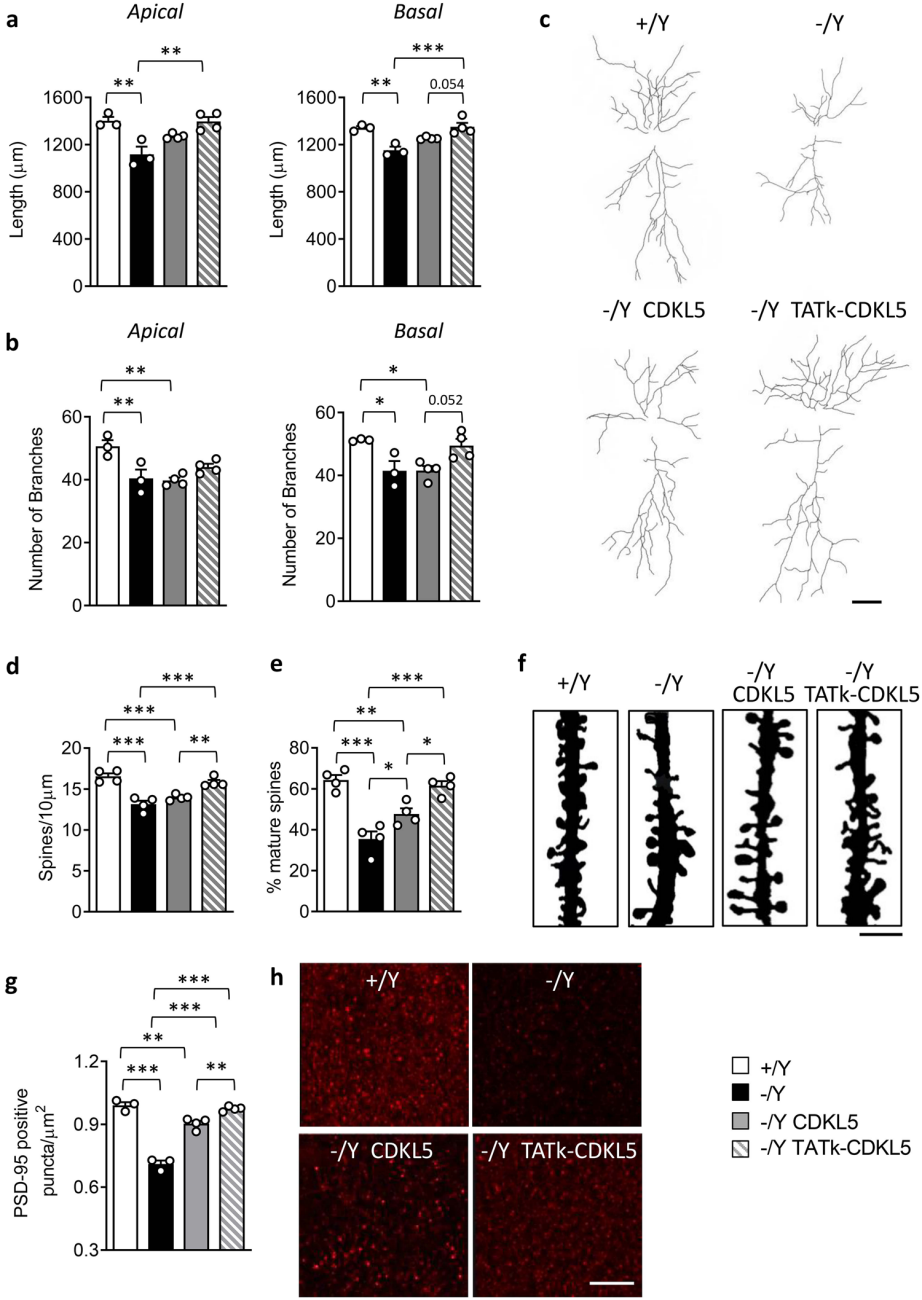
Effect of CDKL5 and TATk-CDKL5 proteins on dendritic morphology and connectivity. **a**, **b** Apical and basal mean total dendritic length (**a**) and mean number of dendritic segments (**b**) of Golgi-stained CA1 pyramidal neurons of wild-type (+ /Y, *n* = 3) and *Cdkl5* − /Y (*n* = 3) mice and of *Cdkl5* − /Y mice 90 days from treatment with AAVPHP.B_CDKL5 (*n* = 4) or AAVPHP.B_Igk-TATk-CDKL5 (*n* = 4). **c** Example of the reconstructed apical and basal dendritic tree of Golgi-stained CA1 pyramidal neurons of 1 animal from each experimental group. Scale bar = 50 µm. **d, e** Dendritic spine density (**d**) and percentage of mature spines in relation to the total number of protrusions (**e**) of CA1 pyramidal neurons from wild-type (+ /Y, *n* = 3) and *Cdkl5* − /Y (*n* = 3) mice and of *Cdkl5* − /Y mice treated with AAVPHP.B_CDKL5 (*n* = 4) or AAVPHP.B_Igk-TATk-CDKL5 (*n* = 4). **f** Images of Golgi-stained dendritic branches of CA1 pyramidal neurons of 1 animal from each experimental group. Scale bar = 2 µm. **g** Number of fluorescent puncta per µm^2^ exhibiting PSD-95 immunoreactivity in the CA1 layer of the hippocampus of wild-type (+ /Y, *n* = 3) and *Cdkl5* − /Y (*n* = 3) mice and of *Cdkl5* − /Y mice treated with AAVPHP.B_CDKL5 (*n* = 4) or AAVPHP.B_Igk-TATk-CDKL5 (*n* = 4). **h** Representative fluorescence image of PSD-95 immunoreactive puncta in the hippocampus of 1 animal from each experimental group. Scale bar = 6 µm. Values are represented as means ± SE. *P < 0.05; **P < 0.01; ***P < 0.001 (Tukey’s test after one-way ANOVA)

Dendritic arborization was found to be reduced in cortical and hippocampal pyramidal neurons of *Cdkl5* − /Y mice [13, 16, 36, 37]. In addition, *Cdkl5* − /Y mice exhibit a deficit in dendritic spine structure and stabilization [11, 16, 36–38] and a reduction in the number of PSD-95-positive puncta [22, 37], which indicates loss of excitatory synaptic contacts. In order to establish the effect of gene therapy on dendritic pattern, we evaluated the dendritic length and spine density of CA1 pyramidal neurons (Fig. 4). In *Cdkl5* − /Y mice treated with AAVPHP.B_Igk-TATk-CDKL5 vector, the length of both apical and basal dendrites was recovered compared to vehicle-treated and AAVPHP.B_CDKL5-treated *Cdkl5* − /Y mice (Fig. 4a, c), an improvement which may be mainly attributable to a recovery in the number of branches (Fig. 4b, c). Unlike in AAVPHP.B_CDKL5-treated *Cdkl5* − /Y mice, we found a recovery of spine density in *Cdkl5* − /Y mice treated with AAVPHP.B_Igk-TATk-CDKL5 vector in comparison with vehicle-treated *Cdkl5* − /Y mice (Fig. 4d, f). The percentage of mature spines was restored in *Cdkl5* − /Y mice treated with AAVPHP.B_Igk-TATk-CDKL5 vector (Fig. 4e), and it was only partially recovered in *Cdkl5* − /Y mice treated with AAVPHP.B_CDKL5 (Fig. 4e). Similarly, the number of PSD-95 puncta was restored in AAVPHP.B_Igk-TATk-CDKL5 treated *Cdkl5* − /Y mice (Fig. 4g, h), while only a partial improvement was present in *Cdkl5* − /Y mice treated with AAVPHP.B_CDKL5 (Fig. 4g, h).

### Effect of Gene Therapy on Neuronal Survival and Microglia Activation in Cdkl5 − /Y Mice

**Fig. 5:**
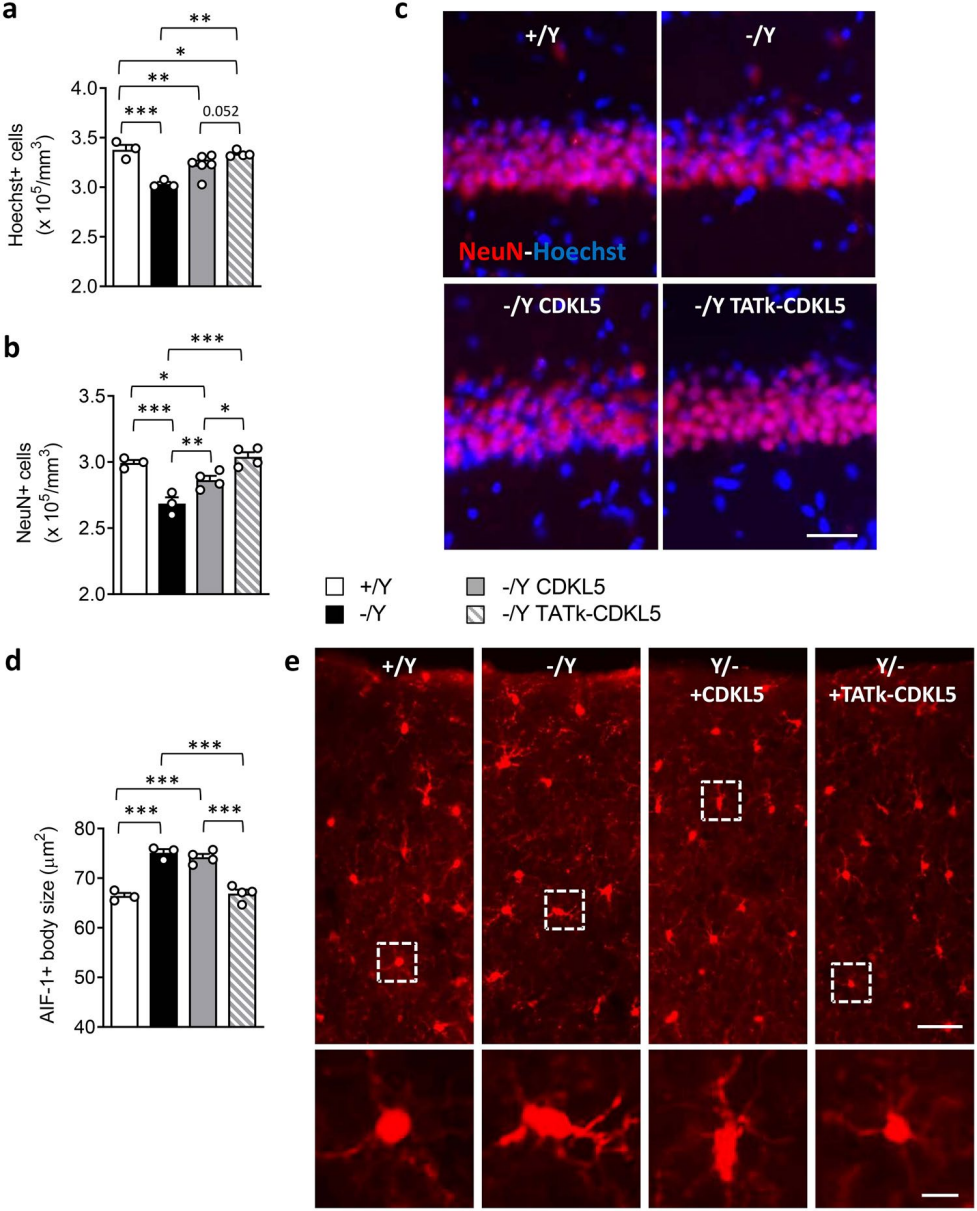
Effect of CDKL5 and TATk-CDKL5 proteins on neuronal survival and microglia activation in the brain of *Cdkl5* − /Y mice. **a**, **b** Quantification of Hoechst-positive cells (**a**) and NeuN-positive cells (**b**) in the CA1 layer of hippocampal sections from wild-type (+ /Y, *n* = 3) and *Cdkl5* − /Y (*n* = 3) mice and from *Cdkl5* − /Y mice treated with AAVPHP.B_CDKL5 (*n* = 4) or AAVPHP.B_Igk-TATk-CDKL5 (*n* = 4). **c** Representative fluorescence images of sections that were immunopositive for NeuN (red) and counterstained with Hoechst (blue) in the hippocampal CA1 region of 1 animal from each group. Scale bar = 50 μm. **d** Mean microglia cell body size in cortical sections from wild-type (+ /Y, *n* = 3) and *Cdkl5* − /Y (*n* = 3) mice, and from *Cdkl5* − /Y mice treated with AAVPHP.B_CDKL5 (*n* = 4) or AAVPHP.B_Igk-TATk-CDKL5 (*n* = 4). **e** Representative fluorescence images of cortical sections processed for AIF-1 immunohistochemistry of 1 animal from each group. The dotted boxes in the upper panels indicate microglial cells shown in magnification in the lower panels. Scale bar = 50 μm (low magnification), 10 μm (high magnification). Values are presented as means ± SE. *P < 0.05; **P < 0.01; ***P < 0.001 (datasets in a and b, Fisher’s LSD test after one-way ANOVA; dataset in d, Tukey’s test after one-way ANOVA)

*Cdkl5* − /Y mice are characterized by decreased survival of hippocampal neurons [39, 40], and by increased microglial activation [41]. *Cdkl5* − /Y mice treated with AAVPHP.B_Igk-TATk-CDKL5 vector showed a higher number of Hoechst-positive nuclei and NeuN-positive pyramidal neurons in the CA1 layer (Fig. 5a–c) in comparison with vehicle- or AAVPHP.B_CDKL5-treated *Cdkl5* − /Y mice, indicating that gene therapy with Igk-TATk-CDKL5 has a greater positive impact on the impaired neuronal survival that is due to loss of Cdkl5 expression. Similarly, a reversal of the inflammatory status, with a reduction in microglial soma size compared to the control levels, was present only in *Cdkl5* − /Y mice treated with AAVPHP.B_Igk-TATk-CDKL5 vector (Fig. 5d, e).

### Evaluation of the Efficiency of AAV Vector Transduction and CDKL5 Biodistribution

**Fig. 6:**
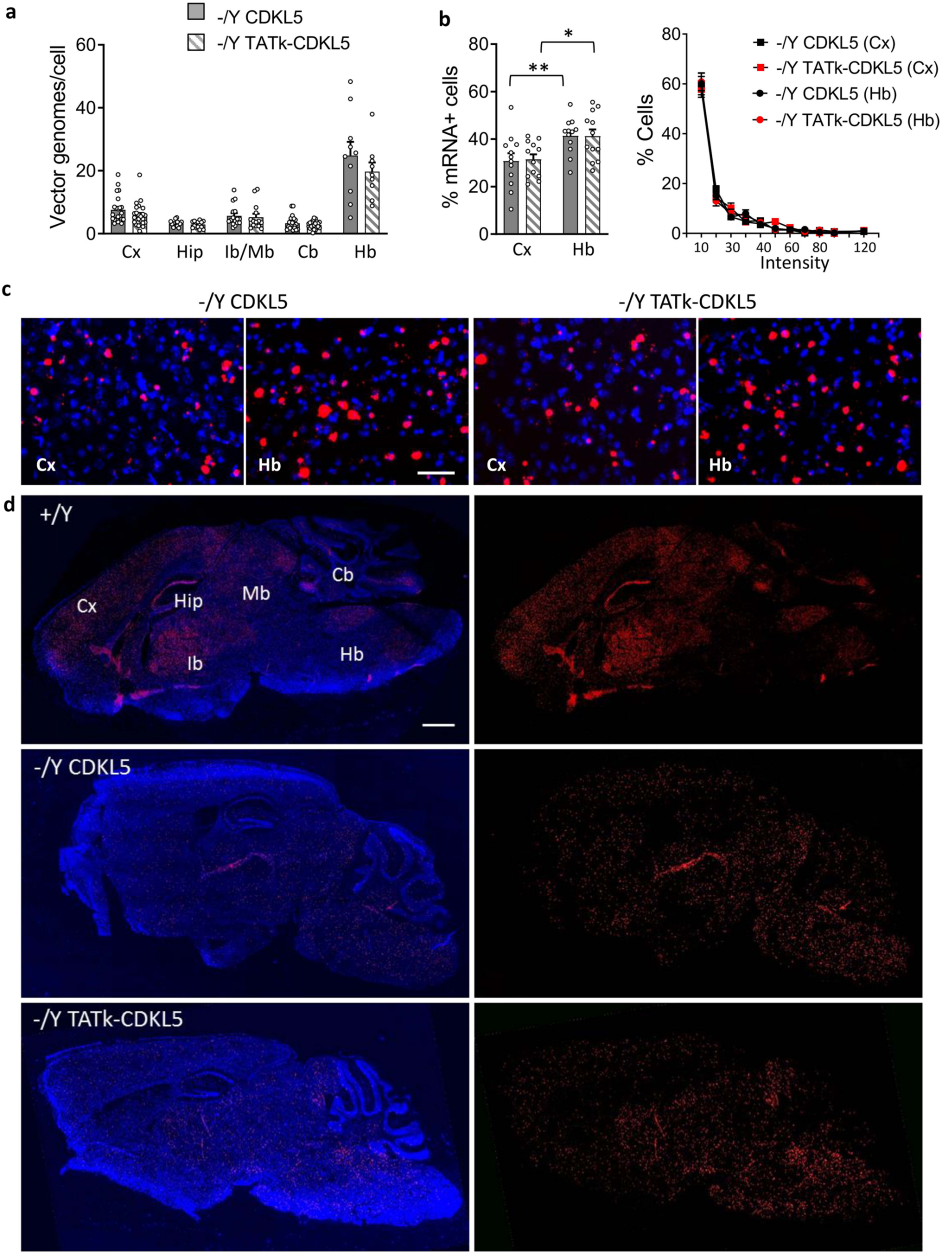
AAV vector transduction and expression and CDKL5 and TATk-CDKL5 proteins in the CNS. **a** Vector genome copy numbers per diploid genomic equivalent in the cortex (Cx, *n* = 24), hippocampus (Hip, *n* = 16–15), midbrain-interbrain (Ib-Mb, *n* = 16–14), cerebellum (Cb, *n* = 23–24), and hindbrain (Hb, *n* = 10–9) of *Cdkl5* − /Y mice treated with AAVPHP.B_CDKL5 or AAVPHP.B_Igk-TATk-CDKL5 according to the treatment schedule shown in Supplementary Fig. 3a. **b** Quantification of the levels of *CDKL5* mRNA expression in the cortex (Cx) and hindbrain (Hb) of *Cdkl5* − /Y mice treated with AAVPHP.B_CDKL5 or AAVPHP.B_Igk-TATk-CDKL5. Left histogram shows the percentage of *CDKL5* mRNA expressing cells, right graph shows the percentage of cells within a specific range of intensity of *CDKL5* mRNA staining. Values are presented as means ± SE in a. Data are given as a percentage of *CDKL5* mRNA expressing cells in b. **c** Representative fluorescence images of cortical (Cx) and hindbrain (Hb) sections, processed for in situ hybridization (ISH) for *CDKL5* mRNA (red) of *Cdkl5* − /Y mice treated as in a. Nuclei were counterstained with Hoechst (blue). **d** Representative sagittal brain sections with fluorescence in situ hybridization (ISH) for *CDKL5* mRNA (red) and nuclei counterstained with Hoechst (blue) of a wild-type mouse (+ /Y) and of an AAVPHP.B_CDKL5 or AAVPHP.B_Igk-TATk-CDKL5 treated *Cdkl5* − /Y mouse. Scale bar = 1000 µm

To quantify and compare the efficiency of gene transfer between AAVPHP.B_Igk-TATk-CDKL5 and AAVPHP.B_CDKL5 vectors, we assessed vector genome copy numbers per cell in several brain regions via qPCR. We found that AAVPHP.B_Igk-TATk-CDKL5 and AAVPHP.B_CDKL5 vectors had the same brain transduction efficiency, regardless of the tropism of various brain regions (Fig. 6a) and induced similar *CDKL5* mRNA levels in the brains of treated mice, evaluated as the number of mRNA-positive cells, mRNA fluorescence intensity per cell, and via qPCR (Fig. 6b–d, Supplementary Fig. 5a). By comparing the *CDKL5* mRNA levels in treated *Cdkl5* − /Y mice to those of wild-type mice, we found that the levels in treated mice were lower than those of wild-type mice in the cortex and hippocampus (Fig. 6d, Supplementary Fig. 5a), indicating only a partial recovery of CDKL5 expression in these brain regions in *Cdkl5* − /Y mice. Only in the hindbrain did the *CDKL5* mRNA reach the levels of wild-type mice, even exceeding them (Fig. 6d, Supplementary Fig. 5a), due to the increased infection efficiency (Fig. 6a), resulting in a higher number of mRNA-positive cells in this brain region (Fig. 6b).

Partial recovery of mRNA expression was confirmed by the low level of CDKL5 expression in the brains of treated *Cdkl5* − /Y mice, evaluated through immunohistochemistry (Fig. 6c, Supplementary Fig. 5b) and Western blot analysis (Supplementary Fig. 5c), reflecting the slight increase in the phosphorylation levels of the direct CDKL5 target, EB2 (Supplementary Fig. 5d, e).

## Discussion

Many chronic neurological diseases do not respond to small molecule therapeutics, and have no effective therapy. Accordingly, no therapies are presently available for the improvement of the neurological phenotypes associated with CDKL5 deficiency disorder. Gene therapy offers the promise of an effective cure for both genetic and acquired brain disease. However, delivering genetic material efficiently to the CNS still remains a hurdle when developing efficacious gene therapy strategies for CNS disorders characterized by widespread neuropathology in several brain regions. By tackling CDKL5 deficiency disorder (CDD) as a model of genetic brain disorder, our study provides novel evidence that a gene therapy approach based on a vector expressing a therapeutic protein fused to an Igk-TATk polypeptide provides increased protein biodistribution and therapeutic efficacy compared to the same vector devoid of the fusion peptide.

Although most CDD patients are females who are heterozygous for CDKL5 deficiency due to random X-chromosome inactivation, hemizygous males have also been reported. The testing of therapies in all genotypes would, of course, be of interest. However, in this proof-of-concept study of gene therapy based on a cross correction mechanism, to avoid the mosaicism interaction that would confound the comparative analysis, we considered *Cdkl5* − /Y mice to be more suitable as an in vivo disease model than *Cdkl5* + */* − mice. Moreover, the age of the mice (adults, 3–4 months) and the route of administration (systemic administration, intracarotid) was chosen to avoid a massive viral infection that would target a high number of cells, potentially hindering, in part, the assessment of effectiveness of the cross-correction mechanism.

We demonstrated that *Cdkl5* − /Y mice treated with AAVPHP.B_Igk-TATk-CDKL5 vector underwent a higher neurodevelopmental and behavioral improvement than mice treated with AAVPHP.B_CDKL5 vector. Importantly, no toxic effects, including immunogenicity problems related to the secreted TATk-CDKL5 protein, were observed in AAVPHP.B_Igk-TATk-CDKL5-treated mice, suggesting the safety of this approach. However, a comprehensive toxicology study needs to be carried out to confirm the safety of this novel gene therapy approach. These promising results suggest that a gene therapy with the Igk-TATk-CDKL5 transgene may be an effective approach to treat CDD. Even if we detected around 6% of cross-corrected cells in the brain of AAVPHP.B_Igk-TATk-CDKL5-treated *Cdkl5* − /Y mice, we believe that the total number of cross-corrected cells was underestimated due to technical detection problems, mainly concerning the difficulty in detecting cells that received a small amount of protein through a cross-correction mechanism. The number is likely to be higher, however, as supported by the many improved outcomes.

Following AAVPHP.B_CDKL5 vector delivery, *Cdkl5* − /Y mice did not exhibit improvement in behavior in comparison with vehicle-treated mice. The poor therapeutic effect of gene therapy with only CDKL5 has previously been reported [42] and was attributed to the necessity of a more robust brain transduction to ameliorate behavioral deficits in this mouse model [43]. It is noteworthy that we found that the secretable TATk-CDKL5 protein led to a higher therapeutic effect compared to CDKL5 alone. With the same infection efficacy as that of the AAVPHP.B_CDKL5 vector, a gene therapy with AAVPHP.B_Igk-TATk-CDKL5 was sufficient to improve various behavioral defects in the *Cdkl5* − /Y mouse, such as innate behaviors and motor performance, and to ameliorate visual function. Albeit in a more marginal way, hippocampal-dependent learning was improved. The lesser therapeutic effect may be attributed to the lower number of viral copies reaching the hippocampus compared to other brain regions. The correlation between levels of CDKL5 re-expression and effectiveness of the gene therapy was confirmed by the finding that in the hindbrain, where the CDKL5 endogenous levels are lower than in the rest of the brain [44], treatment with either AAVPHP.B_CDKL5 or AAVPHP.B_Igk-TATk-CDKL5 vectors led to an higher CDKL5 re-expression than in the other brain regions, as suggested by the mRNA level and protein expression. Accordingly, it is suggestive to note how the centers that regulate the appearance of REM sleep are mainly located in the hindbrain. The high re-expression of CDKL5 in the hindbrain obtained in *Cdkl5* − /Y mice, treated with either AAVPHP.B_CDKL5 or AAVPHP.B_Igk-TATk-CDKL5 vectors, could then explain the normalization of the breathing pattern during REM sleep.

As an anatomical substrate of the ameliorated behavioral performance, in AAVPHP.B_Igk-TATk-CDKL5-treated *Cdkl5* − /Y mice, we found that the impaired dendritic and synaptic development was restored, as was neuronal survival in the hippocampus. By contrast, the lack of behavioral improvement in *Cdkl5* − /Y mice treated with AAVPHP.B_CDKL5 may be accounted for by the reduced effect of treatment on neuronal survival and dendritic development. Similarly, microglia over-activation, a recently described alteration in the brains of *Cdkl5* − /Y mice [41], is inhibited only by treatment with AAVPHP.B_Igk-TATk-CDKL5, further supporting the amplified therapeutic effect of the secretable TATk-CDKL5 protein. The reason why the restoration of several anatomical defects in the hippocampus of AAVPHP.B_Igk-TATk-CDKL5-treated *Cdkl5* − /Y mice does not induce the full recovery of hippocampus-dependent cognitive abilities may be attributable to the complex and intricate in vivo brain function which goes beyond structural restorations.

Interestingly, despite finding a broad distribution of the delivered cassettes over the different brain areas of the viral-injected mice, the viral transcript biodistribution differs to that of the wild-type mice, in which *CDKL5* mRNA is mainly expressed in the cerebral cortex and in the hippocampus. This different distribution could explain the modest therapeutic efficacy shown by the CDKL5 gene therapy. Moreover, we observed unexpected inconsistency between the expression of the viral mRNA and the low CDKL5 protein expression, suggesting a relatively low half-life or reduced translation of the delivered CDKL5. The hypothesis of CDKL5 instability is in agreement with a recent finding showing that the viral *MeCP2* mRNA was not actively translated by ribosomes, underscoring the importance of complex endogenous regulatory elements for MeCP2 protein expression [45]. Similarly, we found that, while the viral *CDKL5* mRNA levels suggest a high CDKL5 re-expression in the brain, i.e., in the hindbrain, the CDKL5 protein level was much lower compared to that found in wild-type mice, indicating that CDKL5 protein levels correlate poorly with viral mRNA levels. Future studies are needed to characterize regulatory elements of the *CDKL5* mRNA and to design a CDKL5 transgene cassette that would be useful to obtain higher CDKL5 protein levels. However, it was demonstrated that even a modest 5–10% re-expression of MeCP2 has a promising therapeutic effect on the Rett Syndrome (RTT) phenotype in a mouse model of RTT [46]. Although the actual quantity of CDKL5 required to achieve therapeutic efficacy is not known, we recently demonstrated, using a protein substitution therapy approach with a TATk-CDKL5 fusion protein [22], that the amount of CDKL5 protein necessary to rescue neurological phenotypes of a mouse model of CDD is very small [22]. Here, we confirmed that the low CDKL5 levels re-expressed in the brains of AAV vector-treated *Cdkl5* − /Y mice are sufficient to ameliorate CDD phenotypes if supported by an increased biodistribution due to the properties of the Igk-TATk fusion protein.

Regardless of the encouraging results in the mouse model, much more needs to be investigated in view of the therapeutic potential of such treatment for CDD patients. First of all, a study on heterozygous females is necessary to verify whether CDKL5 protein production/cross-correction in wild-type cells may lead to an abnormal phenotype. Moreover, a long-term behavioral assessment needs to be performed to demonstrate the efficacy and persistence of transduction over a long time period.

## Conclusions

The improved behavioral performances and neuroanatomical deficits observed in AAVPHP.B_Igk-TATk-CDKL5-treated *Cdkl5* − /Y mice support the amplified therapeutic effect of the secretable TATk-CDKL5 protein compared to CDKL5 alone. Thus, we believe that this study has provided the first proof-of-principle that an innovative gene therapy approach based on the unique advantages of the Igk-TATk fusion peptide is more efficient in improving neurodevelopmental and behavioral impairments in a mouse model of CDD. Such promising results imply that this approach may become a powerful tool for the cure of CDD, and could open avenues to the development of gene therapy for other monogenic diseases based on the unique and compelling properties of the Igk-TATk-fusion protein approach.

## Supplementary Information

Below is the link to the electronic supplementary material.

Supplementary file1 (PDF 508 KB)

Supplementary file2 (PDF 508 KB)

Supplementary file3 (PDF 508 KB)

Supplementary file4 (PDF 533 KB)

Supplementary file5 (PDF 508 KB)

Supplementary file6 (PDF 508 KB)

Supplementary file7 (PDF 508 KB)

Supplementary file8 (PDF 534 KB)

Supplementary file9 (PDF 508 KB)

Supplementary file10 (PDF 508 KB)

Supplementary file11 (DOCX 4804 KB)

Supplementary file12 (PDF 508 KB)

Supplementary file13 (PDF 508 KB)

Supplementary file14 (PDF 551 KB)

Supplementary file15 (PDF 517 KB)

Supplementary file16 (DOCX 39 KB)

Supplementary file17 (DOCX 43 KB)

Supplementary file18 (PDF 526 KB)

Supplementary file19 (PDF 508 KB)

Supplementary file20 (PDF 508 KB)

Supplementary file21 (PDF 508 KB)

Supplementary file22 (PDF 508 KB)

Supplementary file23 (PDF 508 KB)

## Data Availability

Data are available upon request from the corresponding authors.
