# Supplementary material for: Expression of a Secretable, Cell-Penetrating CDKL5 Protein Enhances the Efficacy of Gene Therapy for CDKL5 Deficiency Disorder
Source: Neurotherapeutics. 2022 Sep 15;19(6):1886–904. doi: 10.1007/s13311-022-01295-8 (PMC9723029; doi:10.1007/s13311-022-01295-8)
Supplement: Supplementary file 11 — Supplementary file11 (DOCX 4804 KB) [file 13311_2022_1295_MOESM11_ESM.docx]

**
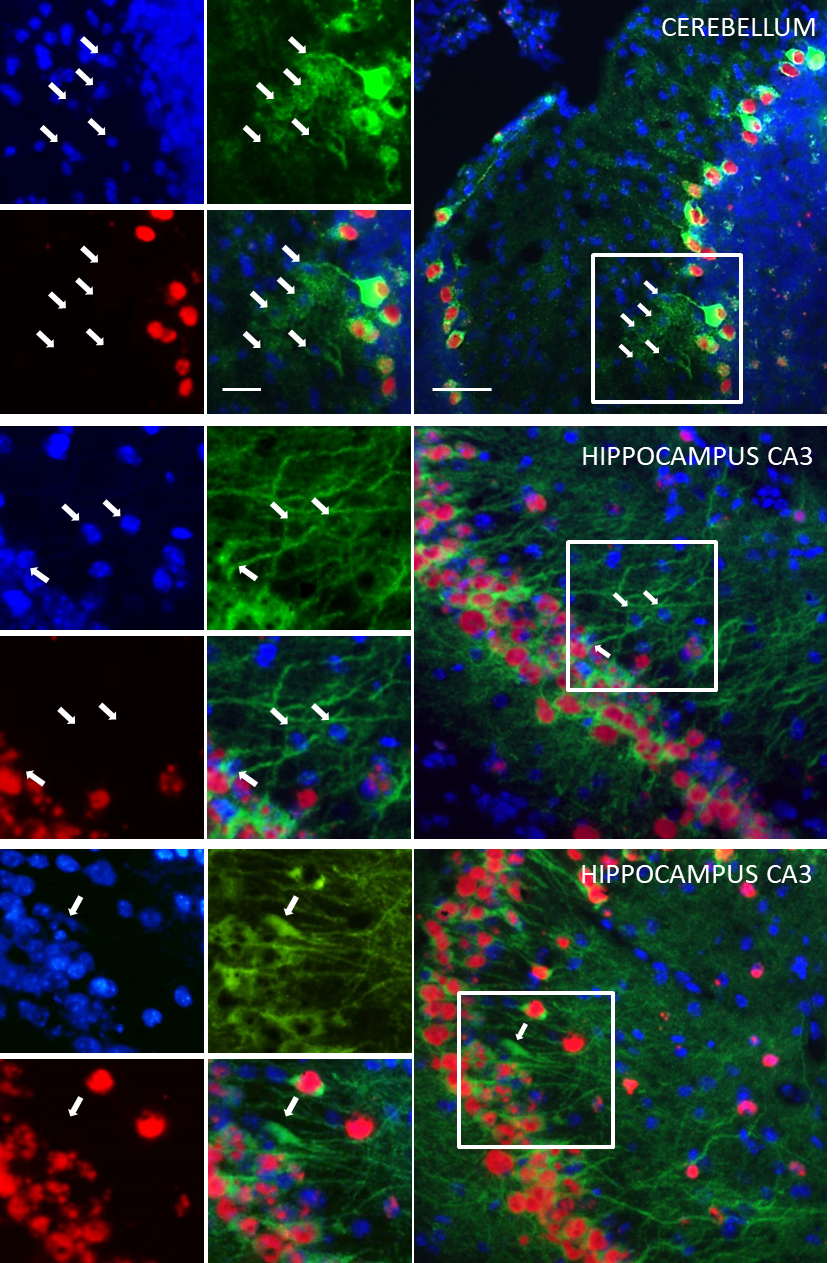
**

**Supplementary Fig. 1**

Fluorescence *In Situ* Hybridization (ISH) for *CDKL5* mRNA combined with fluorescence immunolabeling for TATk-CDKL5 protein in mouse brain sections of 2-month-old *Cdkl5* -/Y mice intraventricularly injected at the neonatal stage with AAVPHP.B_Igk-TATk-CDKL5 vector. Images show *TATk-CDKL5* mRNA (red) and protein (green) localization in the cerebellum and CA3 hippocampal region of a treated mouse 90 days post-injection. Localization of *TATk-CDKL5* mRNA was evaluated through ISH with a CDKL5 probe, while TATk-CDKL5 protein was evaluated through immunohistochemistry using an anti-HA antibody; nuclei were counterstained with Hoechst. The white boxes indicate the regions shown in the high magnification panels. The white arrows indicate cross-corrected cells (HA-immunopositive cells with no ISH staining). Scale bar = 50 µm (low magnification); 25 µm (high magnification).

**
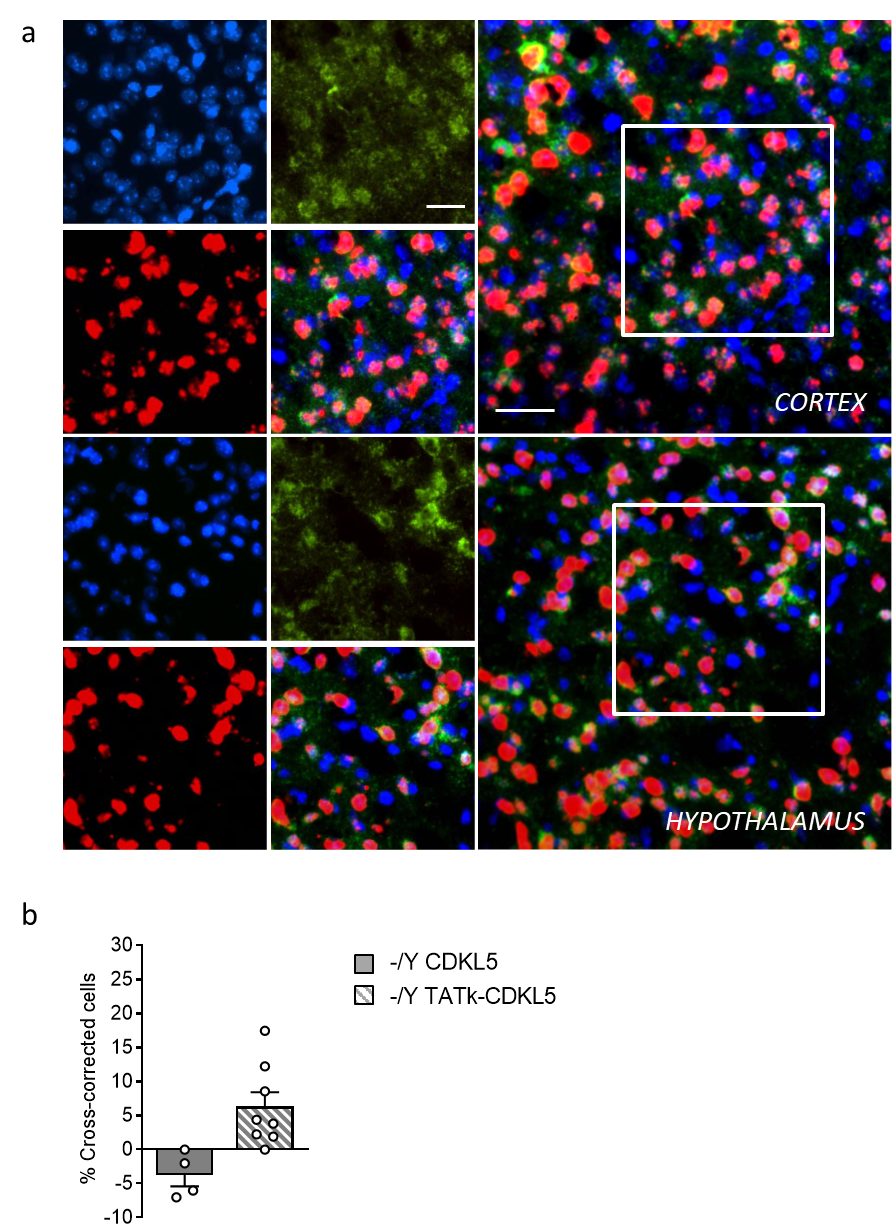
**

**Supplementary Fig. 2**

**(a)** Fluorescence *In Situ* Hybridization (ISH) for *CDKL5* mRNA combined with fluorescence immunolabeling for CDKL5 protein in mouse brain sections of 2-month-old *Cdkl5* -/Y mice intraventricularly injected at the neonatal stage with AAVPHP.B_CDKL5 vector. Images show *CDKL5* mRNA (red) and protein (green) localization in the cortex and hypothalamus of a treated mouse 90 days post-injection. Localization of *CDKL5* mRNA was evaluated through ISH with a CDKL5 probe, while CDKL5 protein was evaluated through immunohistochemistry using an anti-HA antibody; nuclei were counterstained with Hoechst. The white boxes indicate the regions shown in the high magnification panels. Scale bar = 50 µm (low magnification); 25 µm (high magnification). **(b)** Percentage of cross-corrected cells, cells that are positive for CDKL5 protein but not for CDKL5 mRNA, over the number of cells in the hindbrain of treated *Cdkl5* -/Y mice that were positive for CDKL5 mRNA.

**
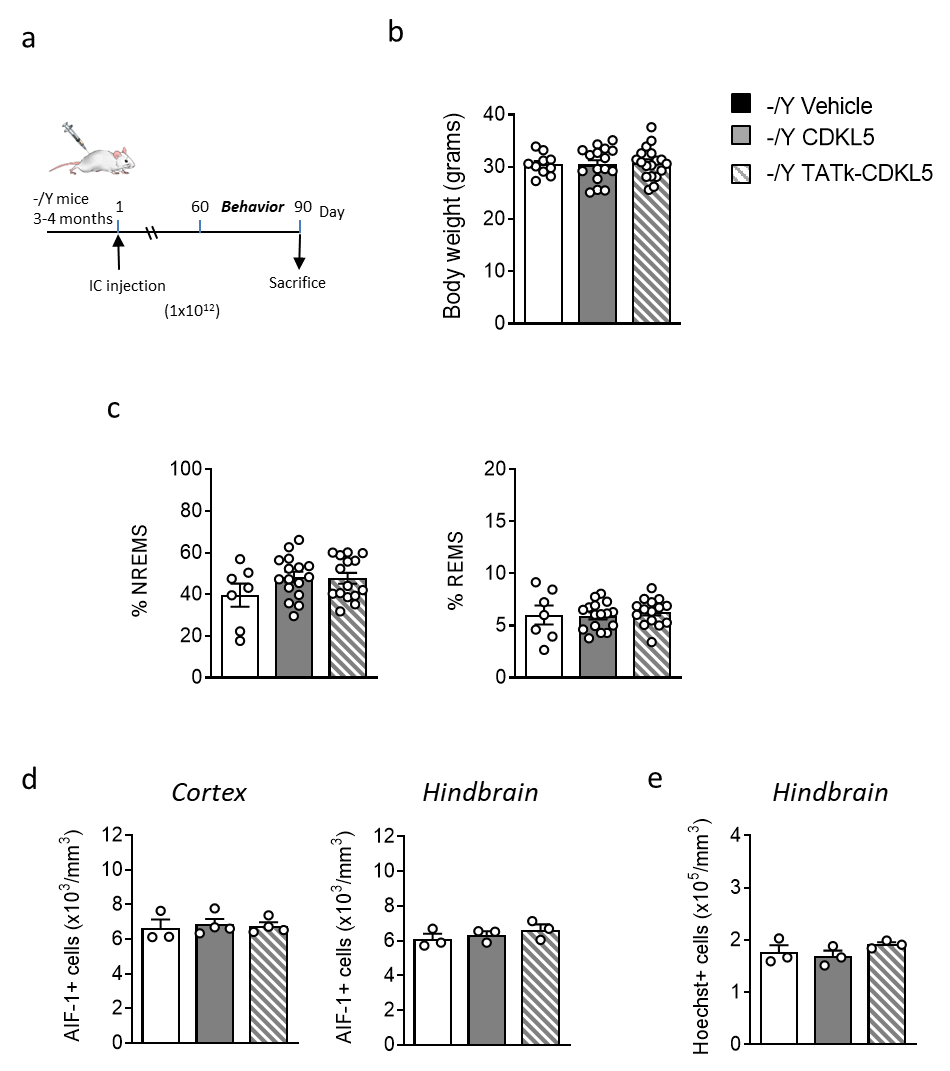
**

**Supplementary Fig. 3**

**(a)** Experimental design. Adult mice (3-4 months old) were systemically treated (intracarotid injection; IC) with vehicle only, AAVPHP.B_CDKL5, or AAVPHP.B_Igk-TATk-CDKL5, and brain samples were collected 90 days post-injection. (**b**) Body weight (in grams) of vehicle-treated *Cdkl5* -/Y mice (-/Y; *n =* 10) and *Cdkl5* -/Y mice treated with AAVPHP.B_CDKL5 (*n =* 16) or AAVPHP.B_Igk-TATk-CDKL5 (*n =* 18) vectors according to the treatment schedule shown in A. Mice were weighed before sacrifice at 90 days post-injection. (**c**) Percentage of time spent in non-rapid-eye-movement sleep (NREMS) and rapid-eye-movement sleep (REMS) during whole-body-plethysmography recordings of vehicle-treated *Cdkl5* -/Y mice (*n =* 8) and *Cdkl5* -/Y mice treated with AAVPHP.B_CDKL5 (*n =* 16) or AAVPHP.B_Igk-TATk-CDKL5 (*n =* 16) vectors. (**d**) Quantification of AIF-1-positive cells in the hippocampus and hindbrain of vehicle-treated *Cdkl5* -/Y mice (*n =* 3) and *Cdkl5* -/Y mice treated with AAVPHP.B_CDKL5 (*n =* 3-4) or AAVPHP.B_Igk-TATk-CDKL5 (*n =* 3-4) vectors. (**e**) Quantification of Hoechst-positive cells in the hindbrain of vehicle-treated *Cdkl5* -/Y (*n =* 3) mice and of *Cdkl5* -/Y mice treated with AAVPHP.B_CDKL5 (*n =* 3) or AAVPHP.B_Igk-TATk-CDKL5 (*n =* 3). Values are presented as means ± SE.

**
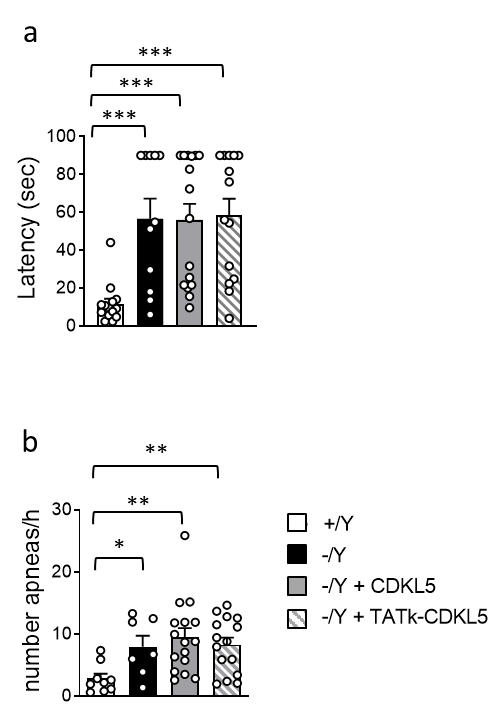
**

**Supplementary Fig. 4**

(**a**) Spatial memory assessed using the Barnes Maze in wild-type mice (+/Y, *n =* 16) and *Cdkl5* -/Y mice (*n =* 10), and in *Cdkl5* -/Y mice treated with AAVPHP.B_CDKL5 (*n =* 15) or AAVPHP.B_Igk-TATk-CDKL5 (*n =* 13). The graph shows the latency to find the target hole on the probe day (day 4). (**b**) Sleep apnea occurrence rate in treated *Cdkl5* -/Y mice was assessed using whole-body plethysmography. Sleep apnea occurrence in vehicle-treated wild-type (+/Y, *n =* 9) and *Cdkl*5 -/Y (*n =* 7) mice, and in *Cdkl5* -/Y mice treated with AAVPHP.B_CDKL5 (*n =* 16) or AAVPHP.B_Igk-TATk-CDKL5 (*n =* 15), during non‐rapid eye movement sleep (NREMS). Values are presented as means ± SE. **P<* 0.05; ***P<* 0.01; ****P<* 0.001 (Dataset in a, Dunn’s test after a Kruskal-Wallis test; dataset in b, Fisher’s LSD test after one-way ANOVA).

**
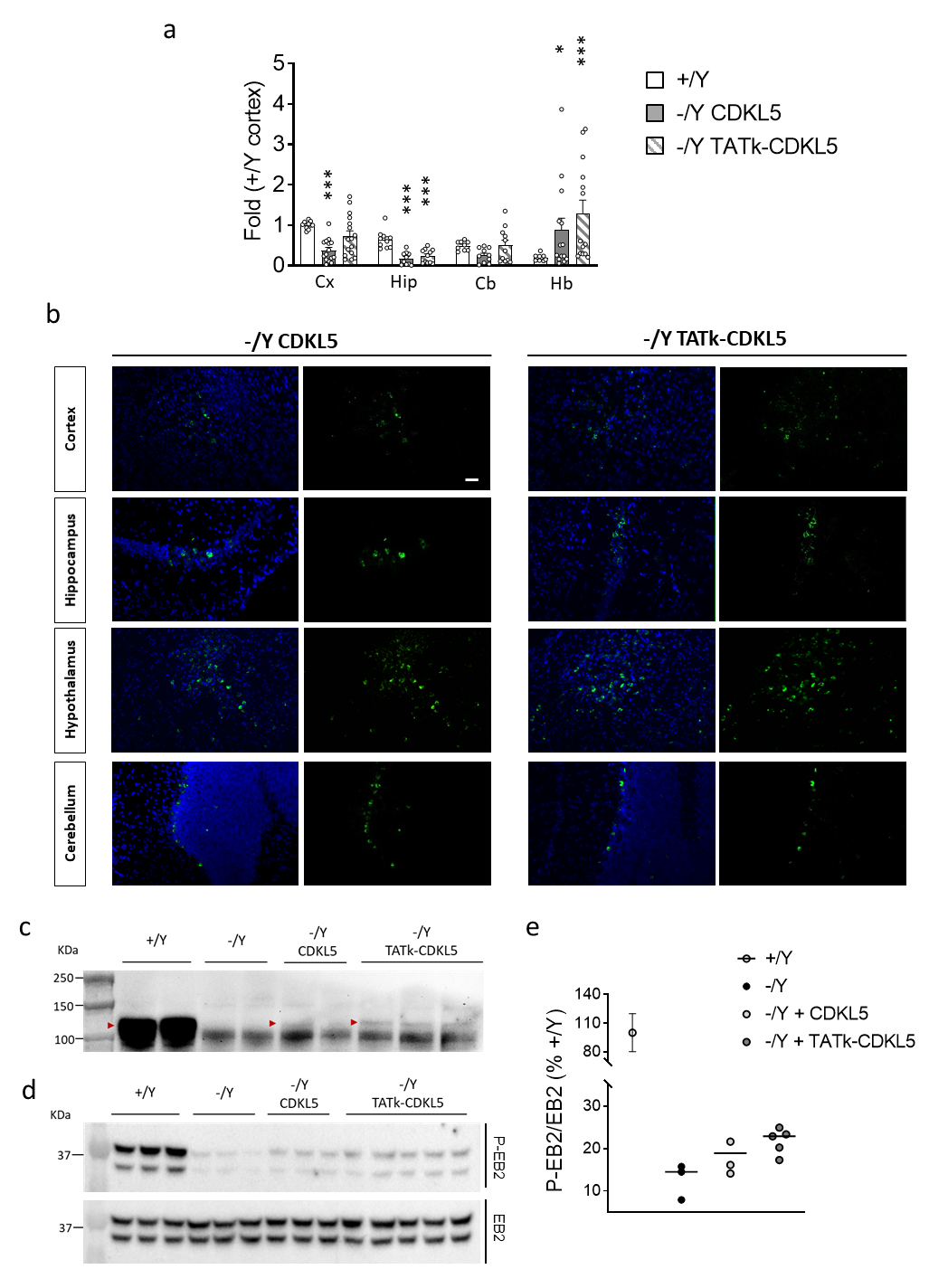
**

**Supplementary Fig. 5**

(**a**) Expression of CDKL5 mRNA in the cortex (Cx, *n =* 10-15-16), hippocampus (Hip, *n =* 10-10-11), cerebellum (Cb, *n =* 9-10-10), and hindbrain (Hb, *n =* 10-14-14) of vehicle-treated wild-type mice (+/Y) and of *Cdkl5* -/Y mice treated with AAVPHP.B vectors (AAVPHP.B_CDKL5 and AAVPHP.B_Igk-TATk-CDKL5). Data are given as the fold change of the +/Y cortex. (**b**) Images show CDKL5 and TATk-CDKL5 protein (green) expression in the cortex, hippocampus, hypothalamus, and cerebellum of a treated mouse 90 days post-injection. CDKL5 and TATk-CDKL5 proteins expression was evaluated through immunohistochemistry using an anti-HA antibody; nuclei were counterstained with Hoechst. Scale bar = 50 µm. (**c**) Western blot analysis of CDKL5 in hindbrain protein extracts from vehicle-treated wild-type (+/Y, *n =* 2) and *Cdkl5* -/Y (*n =* 2) mice, and those from *Cdkl5* -/Y mice treated with AAVPHP.B_CDKL5 (*n =* 2) or AAVPHP.B_Igk-TATk-CDKL5 (*n =* 3) vectors. Red arrowheads indicate mouse Cdkl5 in *Cdkl5* +/Y extracts, human CDKL5, and TATk-CDKL5 in AAVPHP.B_CDKL5 and AAVPHP.B_Igk-TATk-CDKL5 treated *Cdkl5* -/Y mice. (**d,e**) Western blot analysis of phospho-EB2 and total EB2 in cortical protein extracts from vehicle-treated wild-type (+/Y, *n =*3) and *Cdkl5* -/Y (*n =* 3) mice, and those from *Cdkl5* -/Y mice treated with AAVPHP.B_CDKL5 (*n =* 3) or AAVPHP.B_Igk-TATk-CDKL5 (*n =* 5) vectors. Data are expressed as a percentage of +/Y and plotted as dots and median (e). Values in (a) are presented as means ± SE. ****P<* 0.001 compared to the corresponding region of the vehicle-treated wild-type condition (Fisher’s LSD test after one-way ANOVA).
