# Supplementary material for: Expression of a Secretable, Cell-Penetrating CDKL5 Protein Enhances the Efficacy of Gene Therapy for CDKL5 Deficiency Disorder
Source: Neurotherapeutics. 2022 Sep 15;19(6):1886–904. doi: 10.1007/s13311-022-01295-8 (PMC9723029; doi:10.1007/s13311-022-01295-8)
Supplement: Supplementary file 16 — Supplementary file16 (DOCX 39 KB) [file 13311_2022_1295_MOESM16_ESM.docx]

|  | **Target** | **Description** | **Dilution** | **Manufacturer** |
| --- | --- | --- | --- | --- |
| **Primary Antibodies** | AIF-1 | Rabbit polyclonal | IHC 1:300 | ThermoFisher  Cat. n°: PA5-21274 |
|  | CDKL5 | Sheep polyclonal | WB 1:500 | University of Dundee  Cat. n°: SA145 |
|  | EB2 | Rabbit polyclonal | WB 1:1000 | Abcam  Cat. n°: ab234843 |
|  | P-EB2 | Rabbit polyclonal | WB 1:1000 | CovalAb  Cat. n°: pab01032-P |
|  | HA | Rabbit monoclonal | IHC 1:500 ICC 1:200 WB 1:1000 | Cell signalling Technology  Cat. n°: 3724 |
|  | NeuN | Mouse monoclonal | IHC 1:250 | Merck Millipore  Cat. n°: MAB377 |
|  | PSD95 | Rabbit polyclonal | IHC 1:1000 | Abcam  Cat. n°: ab18258 |
|  | βIII Tubulin | Mouse monoclonal | ICC 1:100 | Sigma Aldrich  Cat. n°: T8578 |
| **Secondary Antibodies** | Goat anti-rabbit IgG HRP-conjugated | | WB 1:5000 IHC 1:1000 | Jackson ImmunoResearch  Cat. n°: 111-035-003 |
|  | Donkey anti-sheep IgG HRP-conjugated | | WB 1:5000 | Jackson ImmunoResearch  Cat. n°: 713-035-003 |
|  | Goat anti-mouse IgG FITC-conjugated | | ICC 1:200 | Jackson ImmunoResearch  Cat. n°: 115-095-062 |
|  | Goat anti-rabbit IgG FITC-conjugated | | IHC 1:200 | Jackson ImmunoResearch  Cat. n°: 111-095-045 |
|  | Goat anti-rabbit IgG Cy3-conjugated | | IHC 1:1000 ICC 1:200 | Jackson ImmunoResearch Cat. n°: 111-165-045 |

**Supplementary Table 1.** List of primary and secondary antibodies. WB: western blotting, IHC: immunohistochemistry, ICC: immunocytochemistry.

| **Figure** | **Statistic** | ***P* value** |  | **Figure** | | **Statistic** | ***P* value** |
| --- | --- | --- | --- | --- | --- | --- | --- |
| Fig. 3a | H (3) = 32.57 | *P*<0.0001 |  | Supp. Fig. 4a | | H (3) = 21.97 | *P*<0.0001 |
| Fig. 3b | H (3) = 17.05 | *P=*0.0007 |  | Supp. Fig. 4b | | F (3, 43) = 3.877 | *P*=0.0154 |
| Fig. 3c | H (3) = 19.12 | *P*=0.0003 |  | Supp. Fig. 5a | Ctx | F (2, 38) = 9.231 | *P*=0.0005 |
| Fig. 3d1 | F (3, 82) = 3.000 | *P*=0.0353 |  |  | Hip | F (2, 28) = 18.99 | *P*<0.0001 |
| Fig. 3d2 | F (3, 82) = 2.996 | *P*=0.0354 |  |  | Cb | F (2, 27) = 2.377 | *P*=0.1120 |
| Fig. 3e | F (3, 53) = 9.966 | *P*<0.0001 |  |  | Hb | F (2, 34) = 5.057 | *P*=0.0120 |
| Fig. 3f | H (3) = 48.30 | *P*<0.0001 |  | Supp. Fig. 5b | | F (3, 11) = 20.67 | *P*<0.0001 |
| Fig. 3g | H (3) = 9.543 | *P*=0.0229 |  |  |  |  |  |
| Fig. 3h | F (2, 36) = 2.235 | *P*=0.1217 |  |  |  |  |  |
| Fig. 4a1 | F (3, 10) = 11.2 | *P*=0.0015 |  |  |  |  |  |
| Fig. 4a2 | F (3, 10) = 12.23 | *P*=0.0011 |  |  |  |  |  |
| Fig. 4b1 | F (3, 10) = 10.51 | *P*=0.0020 |  |  |  |  |  |
| Fig. 4b2 | F (3, 10) = 6.553 | *P*=0.0100 |  |  |  |  |  |
| Fig. 4d | F (3, 12) = 23.06 | *P*<0.0001 |  |  |  |  |  |
| Fig. 4e | F (3, 12) = 22.22 | *P*<0.0001 |  |  |  |  |  |
| Fig. 4g | F (3, 10) = 90.54 | *P*<0.0001 |  |  |  |  |  |
| Fig. 5a | F (3, 10) = 13.05 | *P*=0.0009 |  |  |  |  |  |
| Fig. 5b | F (3, 10) = 18.08 | *P*=0.0002 |  |  |  |  |  |
| Fig. 5d | F (3, 10) = 15.17 | *P*=0.0005 |  |  |  |  |  |
| Fig. 6b | F (1,44) = 0.0090 | *P*=0.9246 |  |  |  |  |  |

**Supplementary Table 2.** Descriptive statistic of treatment factor.
